# The GAB-A: Development and Validation of the Gender Stereotypes and Roles Adherence Battery for Adolescents

**DOI:** 10.3390/bs16030413

**Published:** 2026-03-11

**Authors:** Antonio Tintori, Giulia Ciancimino, David Vagni, Loredana Cerbara

**Affiliations:** 1 Institute for Research on Population and Social Policies, National Research Council of Italy, 00185 Rome, Italy; antonio.tintori@cnr.it; 2 Institute for Research and Innovation in Biomedicine, National Research Council of Italy, 90146 Palermo, Italy; david.vagni@irib.cnr.it

**Keywords:** gender stereotypes, gender roles, adolescents, scale development, validation

## Abstract

Validated instruments assessing gender stereotype endorsement among adolescents are scarce and often overlook contemporary domains like digital privacy. To address this gap, this study developed and validated the Gender Stereotypes and Roles Adherence Battery for Adolescents (GAB-A) in a sample of 2955 Italian adolescents attending public secondary schools in Rome (56.4% male; mean age 14.3 years). The battery comprises three modules: the Gender Stereotyped Attitude Scale (GSAS), Gender Role Activities Scale (GRAS), and Gendered Traits Inventory (GTI). Psychometric analysis confirmed robust factor structures, notably identifying a distinct “Relational Control” factor within the GSAS that assesses beliefs normalizing partner surveillance. The results revealed a stark pattern of gender differentiation: males endorsed prescriptive attitudes (GSAS, d = 1.07) and roles (GRAS, d = 0.88) substantially more than females, particularly regarding violence myths. Conversely, essentialist trait beliefs (GTI) showed negligible gender differences (d = 0.11). Associations between stereotypes and psychological health were gender-moderated; within-group analyses indicated that endorsement predicted higher distress, hostility, and alexithymia in males, while being unrelated to well-being in females. Finally, gender-stratified normative data and operational cut-offs were established. The GAB-A provides a psychometrically sound tool for identifying elevated endorsement profiles and evaluating violence prevention interventions.

## 1. Introduction

A stereotype is a cognitive template that perpetuates fixed patterns of thought, generalizing beliefs that oversimplify individual differences. While stereotypes fundamentally constitute false ideas that distort reality, they do not invariably represent negative attributions. For example, Germans may be stereotypically perceived as reliable and punctual, while Southern Italian women might be characterized as excellent cooks—attributions that, although potentially positive, remain preconceived notions divorced from individual variation. More frequently, however, stereotypes function as powerful beliefs that activate prejudicial and/or discriminatory representations of specific social groups.

The first systematic scientific investigation of stereotypes, conducted by Katz and Braly (1933), demonstrated the pervasiveness of internalized prejudices among white college students, who consistently associated African Americans with attributes such as “lazy” and “superstitious,” while characterizing Germans as “rational” and “industrious”. These findings established that stereotypes erroneously attribute specific qualities—predominantly negative—to particular individuals or social groups based on category membership rather than individual characteristics. Gender stereotypes represent perhaps the most pervasive form of social conditioning, emerging through primary socialization processes wherein children internalize what Mead (2015) termed the “generalized other”. Contemporary socialization remains predominantly binary, developing distinct gender-linked cognitive schemas from early childhood (Bem, 1981). Recent studies examining the influence of gender stereotypes confirm that, even when respondents attempt to manage their answers according to social desirability, their self-representations remain clearly shaped by internalized sexism (Cerbara et al., 2022; Lai & Wilson, 2021). Consequently, although younger generations demonstrate greater awareness of gender bias, socialized sexism continues to persist, manifesting in more subtle and implicit forms (Sulla et al., 2025; Moors & De Houwer, 2006). Thus, this binary educational framework continues to shape human existence across the lifespan, generating stereotyped perceptions, expectations, and behaviors transmitted through multiple channels: language acquisition, imitation of emotionally significant adults, media content, educational materials, play activities, and cultural narratives. Research has identified a precise developmental trajectory for prejudice formation in children. By age three, children demonstrate nascent prejudices toward out-groups (Yee & Brown, 1992; Doyle & Aboud, 1995). Prejudicial attitudes emerge by age four, while categorical thinking and in-group/out-group distinctions consolidate between ages five and seven. Between seven and nine years, rigid categorical interpretations begin to attenuate, with more concrete conceptualizations of human differences—particularly regarding sex, age, ethnicity, and group membership—emerging by age eleven (Aboud, 1998).

The persistent prevalence of gender stereotypes across cultures ensures that individuals encounter continuous reinforcement of these beliefs through observable gender roles. These roles establish normative constraints and generate expectations based on biological sex, often associated with differentiated expectations regarding leadership, caregiving, and social authority. The widespread manifestation of these patterns in social reality causes children to perceive stereotypical gender arrangements as natural rather than socially constructed (Berger & Luckmann, 1966). Adolescence represents a particularly critical developmental stage for examining gender stereotypes, as identity consolidation, peer influence, and social comparison processes intensify during this period. Indeed, during adolescence, gender stereotypes increasingly influence educational choices, career aspirations, leadership expectations, and relational norms (Alfieri et al., 1996). Evidence indicates that internalized gender beliefs shape academic self-concept, occupational trajectories, and power dynamics in intimate relationships (Moreau et al., 2021). Measuring stereotype endorsement at this developmental stage is therefore essential for understanding how early cognitive schemas may orient long-term life outcomes. This process establishes a social order mistaken for a natural order, perpetuating itself intergenerationally through binary socialization until the “generalized other” becomes fully integrated into individual consciousness, consolidating gender identity. Identity formation, however, represents an ongoing process subject to continuous remodulation through secondary socialization. Nevertheless, primary socialization experiences constitute the most resistant core of identity structure. Consequently, males progressively assume roles emphasizing dominance, control, and power, while females experience constraint toward—and often internalize—subordinate social positions centered on domestic spheres, caregiving, and partner/offspring nurturance.

Stereotypes thus simultaneously categorize individuals within primary group membership while delineating distinctions from other social groups. Multiple categorization criteria operate: sex, physical appearance, profession, sexual orientation, and social affiliation. Group members share distinctive characteristics that they collectively defend (Tajfel, 1981). Tajfel’s (1986) empirical validation of Mead’s theoretical insights through Social Identity Theory demonstrated how group affiliation mechanisms underpin social discrimination, extending beyond gender to encompass the concepts of race, ethnicity, sexual orientation, and religious affiliation. The fundamental principle operates through ethnocentric bias—perceiving one’s group as superior while devaluing others. When out-groups fail to conform to stereotypically prescribed “natural” standards, discrimination becomes perceived as both justified and inevitable. Gender stereotypes can thus generate discrimination, segregation, and violence.

The propensity to categorize others within meaningful mental containers (sex, ethnicity, religion) while simultaneously self-categorizing derives from stereotypes operating below conscious awareness. Without recognizing these social conditioning processes, individuals enact prescribed social roles while attributing behavior to inherent personality traits or biological predispositions rather than contextual, situational, or cultural factors. This attribution error legitimizes male aggression toward women through perceived superiority and discretionary power, including violence justification. Measuring prejudice presents substantial methodological challenges. Potential discrepancies between questionnaire responses and actual behavior, combined with social desirability bias, complicate accurate assessment (McConahay, 1986). Additionally, individuals may lack awareness of their stereotyped actions (Greenwald & Banaji, 1995). While direct behavioral observation could address these limitations, it lacks the statistical representativeness achievable through large-scale questionnaire administration.

Notable gender-focused instruments include the Bem Sex Role Inventory (BSRI; Bem, 1974) and the Personal Attributes Questionnaire (PAQ; Spence et al., 1975), measuring conformity to traditional gender roles and personality traits associated with gender roles, respectively. However, both instruments employ outdated binary conceptual categories and 1970s-era stereotypes and social norms. The more contemporary Gender Role Stereotypes Scale (GRSS; Mills et al., 2012) offers brevity (eight items) and a distinction between male and female role stereotypes; however, its restricted item pool and emphasis on adult-oriented social roles limit its content validity for adolescent populations. The Ambivalent Sexism Inventory (ASI; Glick & Fiske, 1996), widely utilized internationally, distinguishes hostile from benevolent sexism using an evaluative metric analogous to Pettigrew and Meertens (1995)’ scale. Comprising 22 items equally divided between overt and benevolent/paternalistic sexism measurement, it emphasizes stereotypical thinking over gender role assessment. Given the inherent limitations of any scientific inquiry into abstract constructs, and considering the temporal evolution of gender stereotypes and their impact on life trajectories, attitudes, and behaviors, it is now more imperative than ever to adopt a rigorous methodology capable of identifying both the presence and the nature of gender stereotyping-one that integrates a multidimensional socio-psychometric analysis (Bhatia & Bhatia, 2021; Menegatti et al., 2021; Abele et al., 2016; Hentschel et al., 2019).

Given the limited availability of multidimensional instruments specifically designed to assess gender stereotype endorsement among adolescents, the present study developed the Gender Stereotypes and Roles Adherence Battery for Adolescents (GAB-A).

This battery, employing an interdisciplinary socio-psychological evaluative metric, was implemented in a study of 2955 students attending the first year of public secondary schools of Rome (9th grade; aged 14 years), which was carried out between January and March 2025. The GAB-A is a comprehensive tool comprising three distinct scales: the Gender Stereotyped Attitude Scale (GSAS), which assesses the endorsement of traditional gender stereotypes, the Gender Role Activities Scale (GRAS) that measures beliefs about gender roles and the Gendered Traits Inventory (GTI) that examines attribution of personality characteristics to specific genders. The GAB-A was, therefore, designed to capture three complementary yet distinct components of gender stereotyping among adolescents: prescriptive attitudes, role attributions, and essentialist trait beliefs.

The present study had three primary aims: The first aim was to examine the factorial structure of each scale by testing the robustness of its latent dimensions through a split-sample approach, employing Exploratory Factor Analysis (EFA) in one subsample and Confirmatory Factor Analysis (CFA) in the other. The second aim was to investigate gender differences in endorsement of gender-related beliefs. In this regard, it was hypothesized that male students would report higher endorsement of prescriptive gender stereotypes, as assessed by the GSAS and GRAS, compared to female students. In contrast, no strong directional hypothesis was formulated for essentialist gender trait beliefs, measured by the GTI, and potential differences were explored. The third aim was to assess the psychometric validity of the scales by establishing measurement invariance across gender and school type, thereby ensuring the appropriateness of group comparisons. In addition, convergent validity was examined by testing associations between gender-related beliefs and theoretically relevant constructs, specifically aggressive behaviors and psychological distress. Based on prior literature linking gender stereotype endorsement to psychological adjustment difficulties (Wong et al., 2017; Levant et al., 2009), it was hypothesized that higher GAB-A scores would be positively associated with aggression, psychological distress, hostility, and alexithymia, and negatively associated with self-esteem.

## 2. Materials and Methods

### 2.1. Participants

#### 2.1.1. Recruitment and Sampling

Data were collected as part of the Mutamenti Interazionali e Benessere (MIB; Interactional Changes and Wellbeing) research project, an ongoing longitudinal study investigating social, psychological, and behavioral patterns among Italian adolescents. The MIB study is coordinated by the Mutamenti Sociali, Valutazione e Metodi (MUSA-Social Transformations, Evaluation, and Methods) research group at the Italian National Research Council’s Institute for Research on Population and Social Policies (CNR-IRPPS).

**Figure 1:**
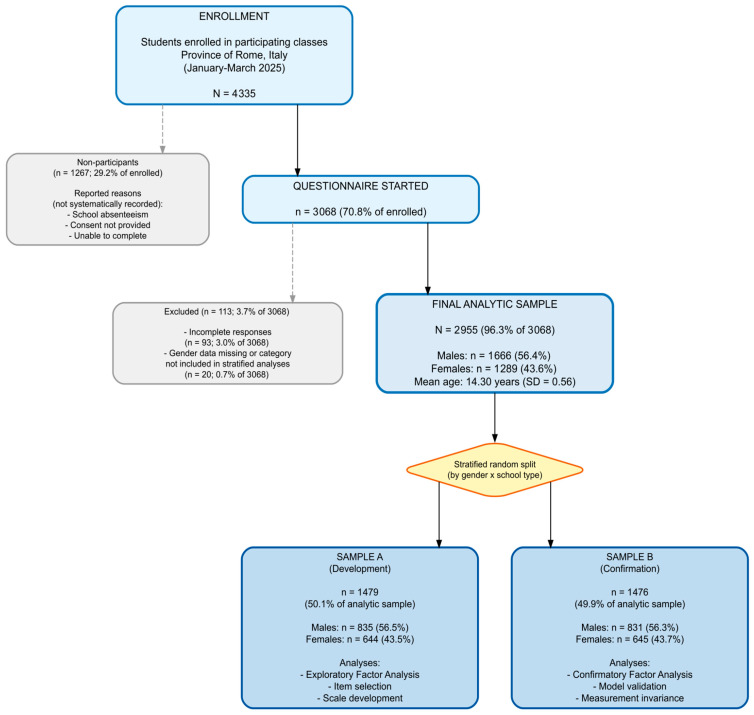
Participant flow and derivation of the analytic samples. Note. Numbers of participants are reported at each study stage (enrolment, participation, exclusions, and analysis), and reasons for non-participation are summarised where available, consistent with STROBE recommendations for observational studies (Item 13). Percentages are computed using the denominator indicated in each box. Gender was self-reported; participants with missing gender data or gender categories not included in the gender-stratified analyses were excluded from the analytic sample. The final analytic sample was randomly split with stratification by gender and school type for cross-validation (Sample A: scale development, exploratory factor analysis; Sample B: confirmation, confirmatory factor analysis, and measurement invariance). The flow-diagram layout follows the widely adopted CONSORT-style convention adapted for non-randomised observational designs.

Participants were recruited from public upper secondary schools located in Rome, which were randomly selected following a cluster sampling design with stratification of primary sampling units to achieve epidemiological representativeness along two stratification dimensions: educational track and geographical location. Based on the 155 urban zones of Rome, the territory was divided into three homogeneous areas differing in the educational level of the resident population. At this first sampling stage, the three areas were identified according to the percentage of residents holding a tertiary education degree, as derived from the 2021 national census. Specifically, areas were categorized as high (more than 30% of residents with a tertiary education degree; 45 urban zones), medium (21–30%; 41 urban zones), and low (up to 20%; 69 urban zones). For each of these three areas, nine schools were randomly selected, three for each school type: vocational institutes, technical institutes, and lyceums (academic). This stratification was adopted because the educational track in Italy is strongly associated with socioeconomic background and may influence attitudes toward gender roles and stereotypes (OECD, 2023). School selection was based on official lists provided by the Italian Ministry of Education and Merit (MIM), resulting in a total of 25 schools rather than 27, as two institutions offered both vocational and technical tracks within the same institution. Within each selected school, during the 2024–2025 academic year, only first-year classes were surveyed, as the study follows a longitudinal design tracking students from the first to the fifth year of upper secondary education. Of the 4335 enrolled students across participating classes, 3068 (70.8%) initiated and completed the questionnaire. Non-participation was attributable to several factors. School absenteeism in Italy during the winter months typically ranges from 20% to 30%, primarily due to seasonal illnesses. Fewer than 5% of parents and/or students declined informed consent. Given Italy’s inclusive education policy, which mandates integration of students with disabilities into mainstream classrooms, a portion of enrolled students may have been unable to complete the questionnaire due to cognitive or learning disabilities. Following data cleaning procedures, 93 cases (3.0%) were excluded due to incomplete responses. An additional 20 participants (0.7%) were excluded because they either did not report their gender or identified as a gender other than male or female (see Figure 1).

#### 2.1.2. Procedures

The survey was conducted between January 2025 and March 2025 using the Computer-Assisted Personal Interviewing (CAPI) method. Each participating school formally signed an Interinstitutional Scientific Research Commitment Agreement to ensure completion of the research project, and informed consent was obtained from all participants’ legal guardian(s), and students were informed about the anonymous and voluntary nature of their participation. The study received approval from the Research Ethics and Integrity Committee of the Italian National Research Council on 13 January 2025. The electronic questionnaire was administered in the presence of trained members of the research team to ensure field control and data quality. This procedure allowed respondents full autonomy in selecting their answers, facilitated comprehension of the questions, and minimized mutual interference among students. Moreover, it helped limit teachers’ involvement during data collection, thereby reducing the risk of bias associated with their presence. The semi-structured questionnaire comprised 90 items covering multiple dimensions of analysis, including socio-economic family background, demographic characteristics, family climate, lifestyle and leisure activities, peer interactions, social media use, online behaviors and experiences, social deviance, stereotypes, risk behaviors, opinions and values, relational and institutional trust, individual and relational well-being.

#### 2.1.3. Sample

Sample characteristics are presented in Table 1. The mean age was 14.30 years (SD = 0.56, range = 13–16). The sample included 1666 males (56.4%) and 1289 females (43.6%).

This gender distribution results from the stratified sampling design, which intentionally oversampled Technical and Vocational tracks to ensure adequate statistical representation despite their lower prevalence in the municipality of Rome. Since these tracks historically attract a higher proportion of male students, balancing the sample across school types consequently increased the male presence relative to the general population.

A chi-square test revealed a significant association between gender and school type, χ^2^(2) = 56.27, *p* < 0.001, Cramér’s V = 0.14. Female students were overrepresented in academic schools (52.4% of females vs. 42.9% of males) and underrepresented in technical institutes (26.1% vs. 39.2%). This pattern reflects documented gender differences in Italian educational tracking, where females disproportionately pursue academic pathways while males more frequently enroll in technical programs (Istat, 2025).

Throughout this paper, the terms ‘male’ and ‘female’ are used to refer to the self-reported gender categories used in the survey instrument, acknowledging that these terms may conflate sex and gender constructs (van Anders, 2015).

Participants were distributed across three school tracks: academic (n = 1390, 47.0%), technical (n = 989, 33.5%), and vocational (n = 576, 19.5%). The majority held Italian citizenship (n = 2656, 89.9%), with 299 students (10.1%) reporting foreign citizenship. Most participants had siblings (n = 2412, 81.6%), while 543 (18.4%) were only children. Parental educational attainment was relatively balanced: low (n = 699, 23.7%), medium (n = 986, 33.4%), medium-high (n = 526, 17.8%), and high (n = 738, 25.0%), with 6 cases (0.2%) missing. Household income showed concentration in the middle category: low (n = 446, 15.1%), medium (n = 1481, 50.1%), and high (n = 1014, 34.3%), with 14 cases (0.5%) missing. Self-reported academic performance was predominantly medium (n = 2024, 68.5%), with 632 students (21.4%) reporting high performance and 299 (10.1%) reporting low performance. No significant gender differences emerged for household income, χ^2^(2) = 5.48, *p* = 0.065, parental education, χ^2^(3) = 5.94, *p* = 0.114, or academic performance, χ^2^(2) = 2.07, *p* = 0.355.

#### 2.1.4. Split-Sample Cross-Validation Design

For construct validity analyses, a split-sample cross-validation strategy was employed to avoid overfitting and ensure generalizability of factor structures (Brown, 2015). The sample was randomly divided into two halves using stratified random sampling by gender and school track: Sample A (n = 1479) for exploratory factor analysis (EFA) and scale development, and Sample B (n = 1476) for confirmatory factor analysis (CFA) and replication. Chi-square tests and independent-samples *t*-tests confirmed equivalence between the two samples on all stratification and demographic variables (Table 2). Gender distribution was identical across samples (43.6% female), χ^2^ < 0.001, *p* > 0.999. Mean age differed by less than 0.04 years (Sample A: M = 14.32; Sample B: M = 14.28), t(2953) = 1.85, *p* = 0.064. School type distribution was equivalent, χ^2^ = 0.001, *p* = 0.999. No significant differences emerged for citizenship, χ^2^ = 1.17, *p* = 0.280, parental education, χ^2^ = 3.72, *p* = 0.293, household income, χ^2^ = 0.86, *p* = 0.650, or academic performance, χ^2^ = 0.91, *p* = 0.635.

### 2.2. Measures

#### 2.2.1. Gender Stereotypes and Roles Adherence Battery for Adolescents (GAB-A)

GAB-A is a multi-module assessment tool developed by the MUSA research group to measure endorsement of gender stereotypes and traditional gender roles among adolescents. The battery comprises three conceptually distinct scales assessing different facets of gender-related beliefs. Full item content and translations are provided in Supplementary Tables S81–S83.

**Gender Stereotyped Attitude Scale (GSAS)**. The GSAS consists of 18 items (17 retained after validation; see Results) assessing endorsement of traditional gender stereotypes through statements about gender differences in abilities, roles, behaviors, and relationship dynamics. Items cover domains including domestic responsibilities (e.g., “It is right that the woman should take care of the house”), professional aptitudes (e.g., “Men have greater leadership ability than women”), emotional characteristics (e.g., “Women are emotionally more fragile than men”), attitudes toward intimate partner violence (e.g., “Intimate partner violence is a private matter that others should not interfere with”), and beliefs about partner surveillance (e.g., “It is right for a man to check his partner’s phone”). Respondents indicate their agreement using a 4-point Likert scale (1 = Strongly disagree; 2 = Somewhat disagree; 3 = Somewhat agree; 4 = Strongly agree). Items are scored so that higher values indicate greater stereotype endorsement. Based on factor analytic results (see Results), the final 17-item GSAS yields a total score and three subscale scores: Traditional Stereotypes (GSAS-TS; 9 items), Violence and Sexuality Myths (GSAS-VM; 5 items), and Relational Control (GSAS-RC; 3 items).

**Gender Role Activities Scale (GRAS)**. The GRAS comprises 18 items (14 retained after validation; see Results) measuring beliefs about gender-based aptitudes for specific activities across domestic, professional, and recreational domains. Activities include cooking, financially supporting the family, childcare, cleaning, sports (football in translated version, calcio in Italian, due to cultural specificity, combat sports, dancing), leadership roles (being in charge at work, being president), earning money, grocery shopping, playing video games, reading books, and police work. Respondents indicate whether they believe males, females, or either gender is better suited for each activity using a three-option categorical response format (Males/Females/It doesn’t matter). Responses were recoded using Schema A to create a stereotype endorsement score: 2 = stereotypical response (matching the traditional gender expectation for that activity), 1 = egalitarian response (“It doesn’t matter”), and 0 = counter-stereotypical response. The stereotypical direction for each item (male or female-associated) was first established based on theoretical expectations and then verified empirically (see Supplementary Table S82). Higher total scores indicate greater endorsement of traditional gender roles. The final 14-item GRAS yields a total score and two subscale scores: Leisure Activities (GRAS-LA; 5 items) and Social Roles (GRAS-SR; 9 items).

**Gendered Traits Inventory (GTI).** The GTI includes 10 items examining attribution of personality characteristics to specific genders, assessing essentialist beliefs about “natural” sex-based personality differences. Traits include independence, aggressiveness, selfishness, self-confidence, sensitivity, reserve, unpredictability, fragility, cooperativeness, and reasonableness. The response format mirrors the GRAS (Males/Females/It doesn’t matter) (Table 3). Critically, responses were recoded using a specific schema (Schema B), which differs from the GRAS: 2 = stereotypical response, 1 = counter-stereotypical response, and 0 = egalitarian response. The rationale for Schema B is that for personality trait attribution, responding “It doesn’t matter” represents a complete rejection of essentialist gender differentiation rather than a neutral intermediate position, whereas a counter-stereotypical response still reflects engagement with gender essentialism. This scoring approach substantially improved internal consistency (see Results). The stereotypical direction for each trait was determined empirically, as two items (Unpredictability and Reasonableness) showed patterns opposite to classical theoretical expectations (Williams & Best, 1990). The GTI yields a single total score; it is unidimensional and has no subscales. Full scoring algorithms for all scales are documented in the GAB-A Scoring Manual (Supplementary Materials: Files S10–S13).

Item development followed a sustained, iterative process spanning approximately ten years of research on gender attitudes in Italian adolescent samples within the same socio-educational context. Items were generated from three sources: (a) adaptation of existing validated measures of gender attitudes and roles, (b) content analysis of contemporary gender-related domains identified through literature review and expert consultation, and (c) novel items addressing emerging domains such as digital privacy and relational surveillance. Individual items were revised multiple times across successive studies based on feedback from adolescent participants, including comprehension difficulties, ambiguous phrasing, and outdated content. All items were drafted following established guidelines for adolescent survey instruments: simple sentence structure, avoidance of double negatives, grade-appropriate vocabulary, and balanced item keying. Sensitive items (particularly those addressing violence myths and sexuality) were reviewed by the ethics committee and by school administrators prior to data collection. Full item content, development rationale, and expert ratings are documented in Supplementary Materials S9 and S10.

For each trait in the GTI, the stereotypical direction was determined empirically using a binomial test comparing the proportion of male-attributed vs. female-attributed responses (excluding egalitarian responses). Items were retained regardless of whether the empirical direction matched classical theoretical expectations, as the goal was to capture contemporary adolescent stereotype content rather than replicate historical patterns.

#### 2.2.2. Measures for Convergent and Discriminant Validity Assessment

**Buss-Perry Aggression Questionnaire (BPAQ).** The Italian adaptation of the 29-item BPAQ (Buss & Perry, 1992; Italian validation: Fossati et al., 2003) was used to assess trait aggression. The scale comprises four subscales: Physical Aggression (9 items), Verbal Aggression (5 items), Anger (7 items), and Hostility (8 items). Items are rated on a 5-point Likert scale (1 = extremely uncharacteristic of me; 5 = extremely characteristic of me). Higher scores indicate greater aggression. In the present sample, internal consistency was excellent for the total score (α = 0.89) and acceptable to good for subscales (Physical: α = 0.82; Verbal: α = 0.68; Anger: α = 0.76; Hostility: α = 0.74). Descriptive statistics for all criterion measures are provided in Supplementary Table S59.

**Kessler Psychological Distress Scale (K10).** The K10 (Kessler et al., 2002; Italian validation: Carrà et al., 2011) is a 10-item screening measure of nonspecific psychological distress experienced in the past 30 days. Items assess symptoms such as feeling tired, nervous, hopeless, restless, and depressed. Responses are provided on a 5-point scale (1 = none of the time; 5 = all of the time), with total scores ranging from 10 to 50. Higher scores indicate greater psychological distress. Internal consistency in the present sample was excellent (α = 0.89).

**Rosenberg Self-Esteem Scale (RSES).** The RSES (Rosenberg, 1965; Italian validation: Prezza et al., 1997) is a widely used 10-item measure of global self-worth. Items assess positive and negative feelings about the self (e.g., “I feel that I have a number of good qualities”; “At times I think I am no good at all”). Responses are provided on a 4-point Likert scale. After appropriate reverse-coding, higher scores indicate higher self-esteem. Internal consistency was good (α = 0.86).

**Perth Alexithymia Questionnaire—Short Form (PAQ-S).** The PAQ-S (Preece et al., 2018) is a brief measure assessing difficulties in identifying and describing emotions. The PAQ-S was developed using a bifactor 6-item self-report framework that yields a more precise measurement of the core alexithymia construct. Responses are provided on a 7-point Likert scale (1 = does not describe me at all; 7 = describes me exactly). Higher scores indicate greater alexithymia. Internal consistency was good (α = 0.79).

### 2.3. Data Analysis

#### 2.3.1. Content Validity Assessment

Content validity was assessed following established psychometric guidelines (Lynn, 1986; Polit & Beck, 2006). A panel of 12 experts was assembled to evaluate the relevance and appropriateness of each item in measuring adherence to gender stereotypes and roles among adolescents. The multidisciplinary panel included clinical psychologists specializing in gender issues (n = 8), occupational health professionals (n = 1), legal professionals with expertise in gender-based violence (n = 1), and academic social psychologists (n = 2). Panel demographics are provided in Supplementary Table S1. This sample size exceeds the minimum recommendation of 6 experts (Lynn, 1986) and aligns with the optimal range of 10–15 experts suggested by Polit and Beck (2006).

Each expert received a formal invitation explaining study objectives and methodology, along with the complete 46-item battery. Experts rated the effectiveness of each item using a 5-point scale (1 = not relevant; 5 = highly relevant) and provided qualitative feedback. Content validity was quantified using the Content Validity Index (CVI) at item and scale levels. The Item-level CVI (I-CVI) was calculated as the proportion of experts rating each item as relevant (score ≥ 4). Following Lynn’s (1986) criteria, items with I-CVI ≥ 0.78 were considered acceptable when evaluated by more than five experts. The Scale-level CVI (S-CVI/Ave) was computed as the arithmetic mean of all I-CVI values, with values ≥ 0.90 indicating excellent content validity (Polit & Beck, 2006). Complete expert ratings are provided in Supplementary Tables S3–S5. Item-level statistics, scale summaries, and flagged items are reported in Supplementary Tables S6–S8.

#### 2.3.2. Factor Structure

**Exploratory factor analysis**. EFA was conducted on Sample A (n = 1479) to examine the internal structure of each GAB-A scale. Data suitability for factor analysis was assessed using the Kaiser-Meyer-Olkin (KMO) measure of sampling adequacy (criterion: ≥0.60 acceptable, ≥0.80 good) and Bartlett’s test of sphericity (criterion: *p* < 0.05). The number of factors to extract was determined through convergence of multiple criteria: eigenvalue > 1 (Kaiser criterion), scree plot inspection, parallel analysis with 1000 iterations (Horn, 1965), and theoretical interpretability (Supplementary Table S10).

For the GSAS (Likert-scale responses), both maximum likelihood extraction with oblimin rotation and polychoric correlations with principal axis factoring and oblimin rotation were used; the results were comparable. For the GRAS and GTI (ordinal responses), polychoric correlations were employed with principal axis factoring and oblimin rotation. Factor loadings ≥ 0.40 were considered salient; items with loadings < 0.40 on all factors or with substantial cross-loadings (difference < 0.20 between primary and secondary loadings) were candidates for exclusion. Because EFA pattern coefficients can be attenuated with ordinal items having few response categories (Rhemtulla et al., 2012), final item retention decisions integrated multiple criteria: EFA pattern coefficients, CFA standardized loadings (WLSMV), content coverage, and corrected item-total correlations. Gender-specific EFA solutions were examined to assess factor congruence across groups (Supplementary Tables S11–S13), quantified using Tucker’s congruence coefficient (φ ≥ 0.95 indicating factor equivalence; Lorenzo-Seva & ten Berge, 2006; Supplementary Table S14).

**Confirmatory factor analysis**. CFA was conducted on Sample B (n = 1476) to confirm the factor structures identified in EFA. Given the ordinal nature of all scale responses, weighted least squares mean and variance adjusted estimation (WLSMV) was employed using polychoric correlations. Model fit was evaluated using multiple indices following established guidelines (Hu & Bentler, 1999): chi-square statistic, comparative fit index (CFI; acceptable ≥ 0.90, good ≥ 0.95), Tucker–Lewis index (TLI; acceptable ≥ 0.90, good ≥ 0.95), root mean square error of approximation (RMSEA; acceptable ≤ 0.08, good ≤ 0.06) with 90% confidence interval, and standardized root mean square residual (SRMR; acceptable ≤ 0.08). Alternative models (e.g., unidimensional, higher-order) were compared using scaled chi-square difference tests (Satorra & Bentler, 2001). Gender-specific CFA models were also fitted to examine factor loading patterns by group (Supplementary Tables S15–S19).

#### 2.3.3. Reliability

Internal consistency was assessed using multiple coefficients computed separately for Sample A and Sample B to examine cross-sample stability. Cronbach’s alpha (α) with 95% confidence intervals was computed for all scales and subscales. McDonald’s omega (ω) was computed as a more robust estimate of reliability when assumptions of tau-equivalence are violated, along with hierarchical omega (ωh) for multidimensional scales to estimate the proportion of variance attributable to the general factor. Point estimates of ω reported in the text were obtained via the Schmid–Leiman decomposition (Revelle, 2025); additionally, Reliability was evaluated by gender and school type to identify potential differential functioning (Supplementary Table S22). CFA-based ω with 95% bootstrap confidence intervals (1000 resamples, WLSMV estimator) are reported in Supplementary Table S22b. Corrected item-total correlations were examined, with values ≥0.30 considered acceptable (Supplementary Tables S23–S25). Polychoric alpha was also computed (Supplementary Table S26). Inter-subscale correlations were computed to evaluate discriminant validity among factors within each scale (Supplementary Tables S27 and S28). A comprehensive reliability summary across demographic groups is provided in Supplementary Table S29.

#### 2.3.4. Known-Groups Validity

Known-groups validity was assessed by examining differences in GAB-A scores across groups expected to differ based on prior research. Gender differences were tested using Welch’s *t*-test (given unequal sample sizes and potential variance heterogeneity) with Cohen’s d effect sizes and 95% confidence intervals. School type differences were examined using one-way analysis of variance (ANOVA) with partial eta-squared (η^2^p) effect sizes and Games-Howell post hoc comparisons (robust to heteroscedasticity). Effect sizes were interpreted according to conventional benchmarks (Cohen, 1988): small (d = 0.20, η^2^ = 0.01), medium (d = 0.50, η^2^ = 0.06), and large (d = 0.80, η^2^ = 0.14). The interaction between gender and school type was tested using a 2 (Gender) × 3 (School Type) factorial ANOVA.

Finally, to investigate the specific patterns of gender differences within the GTI, item-level analyses were conducted using two-proportion z-tests to compare endorsement rates between male and female respondents. To account for multiple comparisons across the 20 target-item combinations, a Bonferroni correction was applied (α_adj_ < 0.0025).

Although participants were recruited through a cluster sampling design (schools → classes), several design features mitigate potential clustering effects. First, schools were randomly selected following a two-dimensional stratification (educational track × geographical area based on resident educational attainment), which removes a major source of between-school variability from the residual clustering effect. Second, only first-year students were surveyed (January–March of the first academic year), meaning that participants had minimal exposure to the specific culture of their assigned school at the time of data collection, substantially reducing school-level socialization effects. Third, the CAPI administration protocol was fully standardized across all classrooms with trained research team members present, minimizing interviewer and context effects. Individual school identifiers were not collected in order to protect participant anonymity, as required by the ethics committee. To quantify potential clustering effects, the proportion of variance attributable to school type (Academic, Technical, Vocational) was estimated via one-way ANOVA (see Results). Additionally, measurement invariance testing across school types was conducted to verify that psychometric properties are not confounded by school-level differences (Tables 11 and 12).

#### 2.3.5. Convergent and Discriminant Validity

Convergent validity was assessed through Pearson correlations between GAB-A scale scores and theoretically related constructs. Based on literature linking gender stereotypes to aggressive behavior and hostile attitudes (Glick et al., 2002; Parrott & Zeichner, 2003), positive correlations with BPAQ aggression scales were predicted, particularly physical aggression. The GTI was expected to show positive correlations with alexithymia given theoretical links between essentialist beliefs and restricted emotional processing (Levant et al., 2009). Discriminant validity was assessed through correlations with self-esteem (RSES), expected to be weak given conceptual distinctness. Inter-scale correlations among the three GAB-A scales were examined to evaluate the extent to which they measure related but distinct constructs. All *p*-values were adjusted for multiple comparisons using the Benjamini–Hochberg (BH) False Discovery Rate (FDR) procedure. Gender-specific correlation patterns were examined to identify potential moderating effects (Supplementary Tables S46–S58).

Based on meta-analytic evidence from attitudinal–behavioral research, which typically reports small effect sizes in this domain (Richard et al., 2003), correlations between GAB-A scores and criterion measures were expected to be statistically significant but small in absolute magnitude, reflecting limited shared variance (approximately 4–6%). Confidence intervals for all correlations are reported in Supplementary Tables S46 and S47.

#### 2.3.6. Measurement Invariance

Multi-group CFA was conducted to test measurement invariance across gender and school type, following established procedures (Chen, 2007; Putnick & Bornstein, 2016). School-type invariance was tested across three groups (Academic, Technical, Vocational) simultaneously in a single multi-group CFA model. Latent mean differences were estimated using the Academic track as the reference group, with pairwise contrasts (Technical–Academic, Vocational–Academic, Vocational–Technical) reported for completeness. Invariance testing proceeded through three increasingly restrictive levels: configural invariance (same factor structure across groups), metric invariance (equal factor loadings), and scalar invariance (equal item thresholds/intercepts). Model comparison was based on changes in CFI (|ΔCFI| < 0.010) and RMSEA (ΔRMSEA < 0.015) as recommended by Chen (2007), given the sensitivity of chi-square difference tests to large sample sizes. When full invariance was not achieved, partial invariance was explored by sequentially releasing equality constraints on items showing the largest modification indices, with a minimum of 80% of items constrained for meaningful interpretation (Putnick & Bornstein, 2016). For scales achieving at least partial scalar invariance, latent mean differences were estimated with one group (females or academic schools) set as the reference (mean fixed to 0, variance fixed to 1). Detailed invariance testing steps are provided in Supplementary Tables S31–S39.

#### 2.3.7. Response Style Analysis

Response patterns were examined to identify potential acquiescent or extreme responding that could bias results. For the GSAS, response style indices included acquiescence response style (ARS; mean of raw item responses) and extreme response style (ERS; proportion of responses at scale endpoints). For the GRAS and GTI, egalitarian response style (EgalRS; proportion of “It doesn’t matter” responses), stereotypical response style (SRS; proportion of stereotypical responses), and counter-stereotypical response style (CRS; proportion of counter-stereotypical responses) were computed. Problematic response patterns were operationally defined as: (a) zero variance responses (all identical responses across items), (b) straight-lining (always selecting the same column position), and (c) extreme acquiescence (>90% agreement on the GSAS). Sensitivity analyses compared psychometric properties between the full sample and a cleaned sample excluding problematic cases. Response style indices and their definitions are provided in Supplementary Tables S60 and S61.

#### 2.3.8. Normative Data and Operational Cutoffs

Descriptive statistics (means, standard deviations, skewness, kurtosis) were computed for all GAB-A scales and subscales, stratified by gender (Supplementary Table S70). Given the unbalanced gender distribution (43.6% female, 56.4% male), gender-balanced weights (50/50) were applied to compute total sample statistics that are unbiased with respect to gender composition. Because the stratified sampling design resulted in an overrepresentation of males (56.4%), unweighted descriptives would overestimate the population-level stereotype endorsement. The 50/50 weighting ensures that total sample statistics reflect equal contributions from both gender groups, approximating a gender-balanced population. Percentile distributions (P5, P10, P25, P50, P75, P90, P95) were computed for sum scores, separately by gender (Supplementary Table S71).

To establish operational cutoffs for identifying elevated stereotype endorsement, two complementary approaches were used. First, percentile-based cutoffs were established at P75 (Elevated) and P90 (Alert) thresholds (Supplementary Table S72). Second, kernel density estimation (KDE) was applied to identify natural breaks (antimodes) in score distributions, providing empirically derived category boundaries not dependent on distributional assumptions (Supplementary Table S73). The Hartigan dip test was used to assess departure from unimodality. A combined classification system integrating both approaches was developed for the GSAS (Supplementary Table S74), with categories: Low, Low-Medium, High-Medium, Elevated, and Very Elevated. Category distributions by gender are reported in Supplementary Table S75.

#### 2.3.9. Software

All analyses were conducted using R version 4.5.0 (R Core Team, 2025). The psych package version 2.5.3 (Revelle, 2025) was used for EFA with polychoric correlations, parallel analysis, and reliability estimation. The lavaan package version 0.6–19 (Rosseel, 2012) was used for CFA and measurement invariance testing. The effectsize package version 1.0.1 (Ben-Shachar et al., 2020) was used for effect size computation with confidence intervals. Kernel density estimation was performed using the density() function with default bandwidth selection. All significance tests were two-tailed with α = 0.05 unless otherwise specified.

An AI-assisted workflow was employed to support manuscript preparation using Claude Opus 4.5 (Anthropic, 2025). Specific tasks included reviewing R code for errors, cross-referencing supplementary tables, and English language editing grounded on source materials. The authors reviewed, verified, and modified all AI-generated outputs, retaining full responsibility for the accuracy and integrity of the final manuscript.

## 3. Results

### 3.1. Content Validity

All 12 invited experts completed the content validity assessment, yielding a 100% response rate. Expert panel characteristics are presented in Supplementary Table S1. The overall Scale-level Content Validity Index (S-CVI/Ave) was 0.967, substantially exceeding the 0.90 threshold for excellent content validity (Polit & Beck, 2006). At the module level, the GSAS achieved the highest S-CVI of 0.986, followed by the GRAS at 0.977 and the GTI at 0.917 (Table 4).

At the item level, 45 of 46 items (97.8%) met the I-CVI ≥ 0.78 threshold for acceptable content validity. Thirty-five items (76.1%) achieved perfect expert agreement (I-CVI = 1.00). The only item falling below the 0.78 threshold was GTI item 7 (Unpredictability; I-CVI = 0.67), which three experts rated as less effective in assessing gender stereotypes. Complete expert ratings by item are provided in Supplementary Tables S3–S5.

Expert qualitative feedback (Supplementary Table S2) highlighted several themes: potential social desirability effects for overtly prejudicial items, suggestions to use gender neutral activity descriptions rather than role titles for certain GRAS items, and recommendations to verify the conceptual clarity of trait attribution items in the GTI.

Based on these results, no items were excluded solely on the basis of content validity assessment. All 46 items proceeded to empirical validation, with the understanding that factor analysis and reliability analyses would provide additional evidence for item retention or exclusion decisions.

### 3.2. Construct Validity: GSAS (Gender Stereotyped Attitude Scale)

#### 3.2.1. Item Exclusion

One item was excluded from the final GSAS. Item 10 (“It is right for a girl to go out with her friends without her partner’s consent”) was originally designed as a reverse-scored item assessing relationship autonomy beliefs. Although the item achieved an acceptable item-total correlation (r = 0.32), it demonstrated very low communality in EFA (h^2^ = 0.12) and failed to load saliently on any factor (maximum loading = 0.21). The final GSAS comprises 17 items.

#### 3.2.2. Exploratory Factor Analysis

EFA was conducted on Sample A (n = 1479). Data were highly suitable for factor analysis: KMO = 0.94 (excellent) and Bartlett’s test of sphericity, χ^2^(136) = 11,247.8, *p* < 0.001. Parallel analysis, scree plot inspection, and theoretical considerations converged on a three-factor solution, which explained 50.4% of total variance (Table 5). Factor congruence across gender subsamples was excellent (Tucker’s φ ≥ 0.97 for all factors; Supplementary Table S14), supporting factorial equivalence.

Factor 1: Traditional Stereotypes (GSAS-TS) comprises 9 items (35.0% variance) reflecting conventional beliefs about gender roles in domestic, professional, and family contexts, including women’s primary role as homemaker and caregiver, men’s superior leadership abilities, and complementary gender responsibilities. Factor loadings ranged from 0.25 (Item 11: “A man has the duty to protect the woman”) to 0.75 (Item 1: “It is right that the woman should take care of the house”). Three items (GSAS_4, GSAS_11, GSAS_18) showed EFA pattern coefficients below the 0.40 salience threshold (0.36, 0.25, and 0.32, respectively) but were retained based on adequate CFA standardized loadings via WLSMV (0.49, 0.50, and 0.63), acceptable corrected item-total correlations (all ≥ 0.36), and content coverage considerations, as these items capture theoretically important facets of the Traditional Stereotypes domain not adequately represented by the remaining items.

Factor 2: Violence and Sexuality Myths (GSAS-VM) comprises 5 items (9.4% variance) capturing beliefs that minimize, justify, or normalize gender-based violence and sexual coercion. This factor includes victim-blaming attitudes (Item 16: “To avoid sexual harassment, women should not dress provocatively”), privacy norms around intimate partner violence (Item 13: “Intimate partner violence is a private matter that others should not interfere with”), and sexual consent myths (Item 15: “When women say ‘no’ to sex, they actually want to do it”). Pattern coefficients ranged from 0.49 to 0.70.

Factor 3: Relational Control (GSAS-RC) comprises 3 items (6.0% variance) assessing beliefs legitimizing surveillance and monitoring behaviors in romantic relationships, specifically, beliefs that men have the right to access partners’ phones, social media passwords, and location information. Pattern coefficients ranged from 0.51 to 0.79.

The three factors showed substantial positive intercorrelations (rs = 0.47–0.68; Supplementary Table S27), consistent with a higher-order general stereotype endorsement construct. Gender-specific EFA solutions are provided in Supplementary Table S11.

#### 3.2.3. Confirmatory Factor Analysis

The three-factor model demonstrated good-to-excellent fit in the independent Sample B (n = 1476): χ^2^(116) = 523.8, *p* < 0.001; CFI = 0.950; TLI = 0.941; RMSEA = 0.049, 90% CI [0.045, 0.053]; SRMR = 0.037. Standardized factor loadings ranged from 0.42 (Item 13: IPV as private matter) to 0.75 (Items 1 and 7), with all loadings statistically significant (*p* < 0.001). Communalities (h^2^) ranged from 0.22 to 0.67 (Table 5). Gender-specific CFA results are provided in Supplementary Tables S15 and S16.

It is well established that EFA with ordinal indicators having few response categories (3–4 points) produces attenuated factor loading estimates, even when polychoric correlations are used, because the limited number of thresholds constrains the recoverable variance (Rhemtulla et al., 2012). CFA with WLSMV estimation, by contrast, directly models the ordinal measurement structure and yields less biased standardized loadings. In the present data, CFA loadings were systematically higher than the corresponding EFA pattern coefficients (e.g., GSAS Item 11: EFA = 0.25, CFA λ = 0.50; GSAS Item 18: EFA = 0.32, CFA λ = 0.63), consistent with the expected attenuation pattern. Accordingly, items with borderline EFA loadings were retained when CFA loadings were adequate (all ≥ 0.42), corrected item-total correlations were acceptable (all ≥ 0.36), and content coverage warranted inclusion. The one exception was GRAS Item 3 (Cooking, CFA λ = 0.37), retained solely for content validity (see Discussion). Expert content validity ratings (I-CVI ≥ 0.78 for all retained items; Supplementary Table S10) further support these retention decisions.

Latent factor correlations were substantial: GSAS-TS with GSAS-VM, r = 0.68; GSAS-TS with GSAS-RC, r = 0.50; GSAS-VM with GSAS-RC, r = 0.47 (Supplementary Table S19). The high interfactor correlations suggest the possibility of a higher-order structure; however, the three-factor solution was retained given its theoretical utility and the distinct correlational patterns observed for GSAS-RC in validity analyses (see Section 2.3.5).

#### 3.2.4. Reliability

Internal consistency for the total 17-item scale was excellent: α = 0.894, 95% CI [0.887, 0.901]; McDonald’s ω = 0.903; CFA-based ω = 0.934, 95% CI [0.931, 0.939] (Supplementary Tables S22 and S22b). Ninety-five percent confidence intervals for all α (Feldt method) and CFA-based ω (1000 bootstrap resamples) estimates, including gender- and school-type-specific coefficients, are provided in Supplementary Table S22b; all CI widths were narrow (≤0.021 for α, ≤0.015 for ω), confirming precise estimation. Subscale reliabilities were acceptable to good: GSAS-TS (9 items) α = 0.851; GSAS-VM (5 items) α = 0.743; GSAS-RC (3 items) α = 0.738. Reliability was stable across the split samples (Sample A: α = 0.896; Sample B: α = 0.891; Supplementary Table S26) and across demographic subgroups, with all coefficients exceeding 0.85 for the total scale and 0.62 for subscales (Supplementary Table S22). All items showed corrected item-total correlations exceeding 0.35 (range: 0.36–0.68; Supplementary Table S23).

#### 3.2.5. Known-Groups Validity

**Figure 2:**
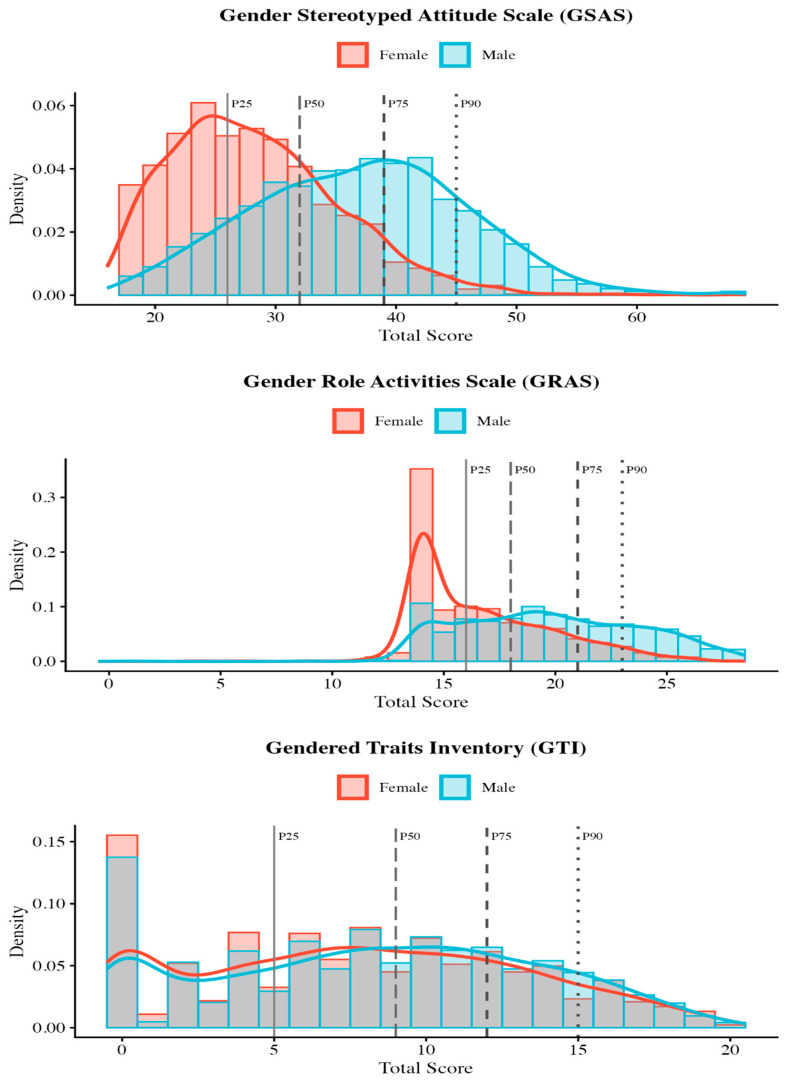
Weighted Score Distributions by Gender and Operational Percentiles for the GAB-A Scales. Note. The figure displays the score distributions for Male (blue) and Female (red) adolescents, relative to the weighted total sample percentiles (dashed vertical lines). GSAS (Attitudes): Higher scores indicate stronger endorsement of traditional stereotypes. GRAS (Roles—Schema A): The distribution is centered around the egalitarian score of 1. Scores < 1 indicate counter-stereotypical behaviors, while scores > 1 indicate adherence to traditional gender roles. GTI (Traits—Schema B): A score of 0 represents the egalitarian (“gender-blind”) response. Higher scores indicate greater gender salience, progressing from counter-stereotypical attribution (1) to traditional stereotype endorsement (2).

**Gender differences**. Males (M = 2.18, SD = 0.53) scored substantially higher than females (M = 1.67, SD = 0.42) on GSAS total scores, t(2953) = 29.71, *p* < 0.001, d = 1.07, 95% CI [0.99, 1.15] (see Figure 2). This large effect was consistent across all three subscales: GSAS-TS (d = 0.95), GSAS-VM (d = 1.04), and GSAS-RC (d = 0.56, medium-large effect; Table 6, Panel A). Item-level gender differences and model-implied expected values are reported in Supplementary Tables S40 and S43.

**School type differences**. One-way ANOVA revealed a significant main effect of school type, F(2, 2952) = 100.85, *p* < 0.001, η^2^ = 0.064 (medium effect; Table 6, Panel B). Post hoc comparisons (Games-Howell) indicated that technical track students (M = 2.12) scored significantly higher than academic track students (M = 1.82; *p* < 0.001), and vocational track students (M = 2.03) also scored higher than academic students (*p* < 0.001). Technical and vocational students did not differ significantly from each other (*p* = 0.057). This pattern was consistent across all three subscales (η^2^ = 0.033–0.056).

**Gender × School Type interaction**. Factorial ANOVA revealed no significant interaction for total scores, F(2, 2949) = 0.39, *p* = 0.680, η^2^p < 0.001, indicating that gender effects were additive across school types (Table 6, Panel C).

**Elevated endorsement profiles**. Males in the technical track showed the highest stereotype endorsement (M = 2.23), while females in the academic track showed the lowest (M = 1.59). The gap between these extreme groups was 0.64 scale points, equivalent to approximately 1.2 standard deviations.

### 3.3. Construct Validity: GRAS (Gender Role Activities Scale)

#### 3.3.1. Item Exclusions

Four items were excluded based on low factor loadings or poor item-total correlations (all < 0.30): Item 1 (Driving; rit = 0.28), Item 4 (Teaching; rit = 0.28), Item 13 (Scientific discoveries; rit = 0.24), and Item 15 (Talking on the phone; rit = 0.29). Documentation of excluded items with rationale is provided in Supplementary Table S84. The final GRAS comprises 14 items.

#### 3.3.2. Exploratory Factor Analysis

EFA was conducted on Sample A using polychoric correlations. Data were suitable for factor analysis: KMO = 0.915 (excellent). Parallel analysis suggested extraction of two factors, which explained 47.9% of variance (Table 7). Factor congruence across gender was excellent (Tucker’s φ ≥ 0.95; Supplementary Table S14).

Factor 1: Leisure Activities (GRAS-LA) comprises 5 items (38.8% variance) capturing gender-stereotyped beliefs about recreational and expressive activities: playing football (Item 7: λ = 0.99), playing video games (Item 14: λ = 0.77), dancing (Item 8: λ = 0.75), practicing combat sports (Item 16: λ = 0.70), and reading books (Item 17: λ = 0.47).

Factor 2: Social Roles (GRAS-SR) comprises 9 items (9.1% variance) assessing beliefs about gendered aptitudes for professional authority, economic provision, and domestic responsibilities. Pattern coefficients ranged from 0.35 (grocery shopping) to 0.79 (earning money). The inter-factor correlation was substantial (r = 0.59; Supplementary Table S28).

#### 3.3.3. Confirmatory Factor Analysis

The two-factor model with WLSMV estimation demonstrated excellent fit: χ^2^(76) = 480.44, *p* < 0.001; CFI = 0.989; TLI = 0.987; RMSEA = 0.055, 90% CI [0.050, 0.059]; SRMR = 0.049. Standardized factor loadings ranged from 0.37 (Cooking) to 0.90 (Playing football; Table 7). The latent factor correlation was r = 0.84. Gender-specific CFA results are provided in Supplementary Table S17.

#### 3.3.4. Reliability

Internal consistency for the total 14-item scale was good: α = 0.850, 95% CI [0.842, 0.858]; McDonald’s ω = 0.837; CFA-based ω = 0.915, 95% CI [0.909, 0.921] (Supplementary Table S22b). Subscale reliabilities were acceptable: GRAS-LA (5 items) α = 0.781; GRAS-SR (9 items) α = 0.783. Reliability was stable across samples (Sample A: α = 0.854; Sample B: α = 0.845; Supplementary Table S26) and demographic subgroups (range: 0.807–0.852 for the total scale). Item-total correlations ranged from 0.31 to 0.58 (Supplementary Table S24).

#### 3.3.5. Known-Groups Validity

**Gender differences**. Males (M = 1.42, SD = 0.28) scored substantially higher than females (M = 1.19, SD = 0.22). on GRAS total scores, t(2953) = 24.55, *p* < 0.001, d = 0.88, 95% CI [0.81, 0.96] (see Figure 2). The effect was largest for Leisure Activities (d = 0.89) and medium-large for Social Roles (d = 0.70; Table 8, Panel A). Item-level gender differences and model-implied expected values are reported in Supplementary Tables S41 and S44.

**School type differences**. One-way ANOVA revealed a significant but smaller effect of school type, F(2, 2952) = 31.89, *p* < 0.001, η^2^ = 0.021 (small effect; Table 8, Panel B). Notably, the pattern differed from the GSAS: technical institute students (M = 1.37) scored higher than both academic (M = 1.29) and vocational (M = 1.29) students, while academic and vocational students did not differ from each other.

**Gender × School Type interaction**. No significant interaction was observed, F(2, 2949) = 2.13, *p* = 0.119, η^2^p = 0.001 (Table 8, Panel C).

### 3.4. Construct Validity: GTI (Gendered Traits Inventory)

#### 3.4.1. Scoring Schema and Empirical Verification

A critical preliminary analysis revealed that the standard scoring approach produced unacceptable internal consistency. Two scoring schemas were compared. Schema A (standard) coded responses as: stereotypical = 2, egalitarian = 1, counter-stereotypical = 0. Schema B (alternative) coded: stereotypical = 2, counter-stereotypical = 1, egalitarian = 0. Schema A yielded α = 0.515, while Schema B produced α = 0.802, an improvement of nearly 0.30 in reliability.

The theoretical justification for Schema B is that for personality trait attribution, responding “it doesn’t matter” (the egalitarian option) represents a complete rejection of essentialist gender differentiation (scored 0), not a neutral intermediate position. In contrast, a counter-stereotypical response (attributing a trait to the non-traditional gender) still reflects engagement with gendered categorization (gendered salience) and is thus scored 1. Schema B was adopted for all GTI analyses.

Raw response distributions were examined to verify which gender is stereotypically associated with each trait in this sample (Supplementary Table S87). Eight of 10 items aligned with classical theoretical expectations from the gender stereotype literature (Williams & Best, 1990). However, two items showed patterns opposite to predictions and required empirical recoding: Item 7 (Unpredictability) was theoretically expected to be associated with females (emotional instability stereotype), but was empirically associated with males (19.5% vs. 14.7% attributing it to males vs. females). Item 10 (Reasonableness) was expected to be associated with males (rationality stereotype) but was empirically associated with females (27.0% vs. 10.2%).

When items 7 and 10 were coded according to theoretical rather than empirical directions, reliability dropped substantially (from α = 0.802 to α = 0.359). The empirically derived stereotype directions were therefore used for all analyses. Documentation of this recoding verification is provided in Supplementary Table S30.

#### 3.4.2. Confirmatory Factor Analysis

CFA was conducted on Sample B. The unidimensional model showed acceptable fit: χ^2^(35) = 321.56, *p* < 0.001; CFI = 0.948; TLI = 0.934; RMSEA = 0.075, 90% CI [0.067, 0.082]; SRMR = 0.063. Standardized factor loadings ranged from 0.53 (Independence) to 0.77 (Sensitivity), with all loadings significant (*p* < 0.001; Table 9). Communalities (R^2^) ranged from 0.28 to 0.60, indicating that between 28% and 60% of item variance was explained by the latent trait factor.

#### 3.4.3. Reliability

Internal consistency was good: α = 0.802, 95% CI [.792, 0.813]; McDonald’s ω = 0.816; CFA-based ω = 0.890, 95% CI [0.883, 0.898] (Supplementary Table S22b). Reliability was stable across samples (Sample A: α = 0.808; Sample B: α = 0.796; Supplementary Table S26) and remarkably consistent across gender (female: α = 0.801; male: α = 0.805) and school type subgroups (range: 0.788–0.831). Item-total correlations ranged from 0.41 to 0.54 (Supplementary Table S25).

#### 3.4.4. Known-Groups Validity

**Gender differences**. In stark contrast to the GSAS and GRAS, gender differences on the GTI were negligible. Males (M = 0.83, SD = 0.53) scored marginally higher than females (M = 0.78, SD = 0.53), t(2953) = 2.94, *p* = 0.003, d = 0.11, 95% CI [0.04, 0.18] (Table 10, Panel A). This effect size is approximately 10 times smaller than those observed for the GSAS (d = 1.07) and GRAS (d = 0.88). The raw difference of 0.05 points represents only 2.5% of the scale range. Item-level gender differences and model-implied expected values are reported in Supplementary Tables S42 and S45.

Despite the negligible aggregate effect size on the GTI, item-level analysis revealed significant bidirectional differences (see Supplementary Table S87). When examining the “Self-Attribution Gap” (Δ; the difference between self-ratings and outgroup-ratings), consistent differences were observed. For positive traits such as Independence and Reasonableness, both gender groups significantly over-attributed the trait to themselves compared to how they were perceived by the outgroup (*p* < 0.001). Conversely, regarding negative traits, female respondents significantly under-attributed Fragility and Unpredictability to themselves compared to outgroup ratings (*p* < 0.001), whereas male respondents over-attributed Unpredictability to themselves (*p* < 0.05).

**School type differences**. A small but statistically significant effect emerged, F(2, 2952) = 4.47, *p* = 0.012, η^2^ = 0.003 (negligible effect; Table 10, Panel B). Post hoc comparisons indicated that technical track students (M = 0.85) scored marginally higher than academic track students (M = 0.78; *p* = 0.012), but the relevance of this difference is minimal.

**Gender × School Type interaction**. No significant interaction was observed, F(2, 2949) = 0.55, *p* = 0.579, η^2^p < 0.001 (Table 10, Panel C).

#### 3.4.5. Measurement Invariance

Multi-group CFA was conducted to test measurement invariance across gender and school type, following Chen’s (2007) criteria: ΔCFI ≥ −0.010 and ΔRMSEA ≤ 0.015 for invariance support. Results are summarized in Table 11 and Table 12.

#### 3.4.6. Invariance Across Gender

**GSAS**. The three-factor model achieved full scalar invariance across gender (Table 11). The configural model showed good fit (CFI = 0.988, RMSEA = 0.045), and imposing metric constraints (ΔCFI = −0.004, ΔRMSEA = +0.005) and scalar constraints (ΔCFI = −0.003, ΔRMSEA = +0.001) resulted in minimal fit degradation. All 17 items functioned equivalently across gender groups, supporting valid latent mean comparisons. The latent mean difference (males higher) was d = 1.07 (Table 11, Panel C).

**GRAS**. The two-factor model achieved full scalar invariance with 14 items (Table 11). During preliminary invariance testing, Item 1 (Driving) showed substantial differential item functioning (DIF), with markedly different loadings across gender (λ = 0.39 for females vs. λ = 0.70 for males). This item was removed from the final scale, and invariance was re-established with the remaining 14 items. Configural to metric (ΔCFI = −0.009) and metric to scalar (ΔCFI = +0.001) transitions met criteria. The latent mean difference was d = 1.06 (20% higher than observed).

**GTI**. The unidimensional model achieved partial scalar invariance (Table 11). Configural (CFI = 0.978) to metric (ΔCFI = −0.001) invariance was supported. However, full scalar invariance failed (ΔCFI = −0.018, exceeding the −0.010 criterion). Examination of modification indices identified two items with threshold non-invariance: Item 1 (Independence) and Item 10 (Reasonableness). After freeing thresholds for these items, partial scalar invariance was achieved (ΔCFI = −0.006 from metric model), with 8 of 10 items (80%) serving as anchor items. The latent mean difference was small (d = 0.14).

#### 3.4.7. Invariance Across School Type

All three scales achieved full scalar invariance across the three school types (Academic, Technical, Vocational), with all ΔCFI and ΔRMSEA values meeting Chen’s (2007) criteria (Table 12). This supports the validity of comparing scale scores across educational tracks without concern for measurement bias.

Latent mean differences by school type (Table 12, Panel C) confirmed the observed score patterns: Technical institute students scored higher than Academic students on all scales (GSAS: d = 0.54; GRAS: d = 0.32; GTI: d = 0.13), while differences between Vocational and Academic students were smaller (GSAS: d = 0.35; GRAS: d = −0.01; GTI: d = 0.02).

### 3.5. Convergent and Discriminant Validity

Correlations between GAB-A scales and criterion measures are presented in Table 13.

#### 3.5.1. Inter-Scale Correlations

The three GAB-A scales showed moderate-to-strong positive intercorrelations, supporting related but distinct constructs (Table 13, Panel B). The GSAS and GRAS showed a strong correlation (r = 0.653, *p* < 0.001), indicating substantial shared variance (43%) while maintaining sufficient uniqueness (57%) to justify separate scales. The GTI correlated moderately with both the GSAS (r = 0.312, *p* < 0.001) and GRAS (r = 0.440, *p* < 0.001). The stronger GTI–GRAS association (compared to GTI–GSAS) suggests that essentialist trait attributions share more conceptual overlap with role beliefs than with explicit attitudinal stereotypes.

Within-scale subscale correlations were substantial but not redundant: GSAS-TS with GSAS-VM (r = 0.68), GSAS-TS with GSAS-RC (r = 0.50), and GSAS-VM with GSAS-RC (r = 0.47). GRAS-LA and GRAS-SR correlated at r = 0.59.

#### 3.5.2. Convergent Validity: Aggression

All three GAB-A scales demonstrated significant positive correlations with physical aggression (BPAQ Physical subscale), providing support for convergent validity (Table 13, Panel A). The GSAS showed the strongest association (r = 0.250, *p* < 0.001), followed by the GRAS (r = 0.187, *p* < 0.001) and GTI (r = 0.178, *p* < 0.001). These correlations remained significant after FDR correction for multiple comparisons.

Gender-stratified analyses (Supplementary Tables S46 and S47) confirmed that the physical aggression association was robust across gender groups. For the GSAS, the correlation was stronger among males (r = 0.271, *p* < 0.001) than females (r = 0.102, *p* < 0.001) but remained significant in both groups. A similar pattern emerged for the GRAS (males: r = 0.218, *p* < 0.001; females: r = 0.016, ns) and GTI (males: r = 0.215, *p* < 0.001; females: r = 0.120, *p* < 0.001).

Analysis of GSAS subscales revealed that GSAS-RC (Relational Control) showed a particularly strong and consistent correlation with physical aggression across both genders (males: r = 0.236, *p* < 0.001; females: r = 0.173, *p* < 0.001). This subscale showed the most robust convergent validity evidence, with significant associations maintained even among females, where other subscales showed weaker effects.

#### 3.5.3. Correlations with Psychological Wellbeing: Gender-Stratified Patterns

Aggregate correlations between GSAS/GRAS scores and psychological wellbeing measures showed an unexpected pattern: negative associations with distress (GSAS: r = −0.149; GRAS: r = −0.184), hostility (GSAS: r = −0.101; GRAS: r = −0.106), and alexithymia (GSAS: r = −0.096; GRAS: r = −0.113), and positive associations with self-esteem (GSAS: r = 0.123; GRAS: r = 0.161; all *p* < 0.001).

Gender-stratified analyses (Supplementary Tables S46 and S47) revealed divergent within-group patterns. Among males, stereotype endorsement showed positive correlations with distress (GSAS: r = 0.066, *p* = 0.007), hostility (r = 0.085, *p* < 0.001), and alexithymia (r = 0.095, *p* < 0.001), and a negative correlation with self-esteem (r = −0.075, *p* = 0.002). Among females, these associations were non-significant (distress: r = −0.046, *p* = 0.10; self-esteem: r = 0.036, *p* = 0.20; alexithymia: r = −0.013, *p* = 0.64) or weakly negative (hostility: r = −0.071, *p* = 0.011).

Two exceptions emerged. The GSAS-RC subscale showed consistent positive associations with distress in both genders (males: r = 0.083, *p* < 0.001; females: r = 0.062, *p* = 0.026). The GTI showed positive correlations with distress in both subgroups (males: r = 0.110, *p* < 0.001; females: r = 0.087, *p* = 0.002). The implications of these gender-moderated patterns are addressed in the Discussion.

#### 3.5.4. Response Style Analysis

Response patterns were examined to identify potential acquiescent responding or other problematic patterns (Supplementary Tables S60–S69). Problematic response patterns were operationally defined as: (a) zero variance responses (all identical responses across items), (b) straight-lining, or (c) extreme acquiescence (>90% agreement on the GSAS).

Rates of problematic responding were very low: GSAS (44 cases, 1.49%), GRAS (1 case, 0.03%), and GTI (5 cases, 0.17%; Table 14). Sensitivity analyses comparing the full sample with a cleaned sample excluding problematic cases revealed minimal impact on psychometric properties. For the GSAS, excluding 44 problematic cases resulted in negligible changes: ΔM = 0.006, Δα = −0.007, Δd = −0.01. The GRAS and GTI showed even smaller changes given the near-absence of problematic patterns.

Response style indices (acquiescence, extreme responding, egalitarian responding) showed weak correlations with scale scores and criterion measures (Supplementary Tables S68 and S69), indicating that GAB-A scores are not substantially confounded by response style artifacts. These results demonstrate the robustness of findings to potential response biases.

### 3.6. Normative Data and Operational Cutoffs

#### 3.6.1. Descriptive Statistics

Descriptive statistics for all GAB-A scales and subscales are presented in Supplementary Table S70. Given the unbalanced gender distribution (43.6% female, 56.4% male), gender-balanced weights (50/50) were applied to compute unbiased total sample statistics.

For the GSAS (range: 1–4), the weighted total sample mean was M = 1.93 (SD = 0.54), with slight positive skewness (0.51) indicating a floor effect. Females showed lower means (M = 1.67, SD = 0.42) with pronounced positive skewness (0.84), while males showed higher means (M = 2.18, SD = 0.53) with near-normal distribution (skewness = 0.14).

For the GRAS (range: 0–2, with 0 representing complete counter-stereotypical responding, 1 representing the egalitarian midpoint, and 2 representing complete stereotypical responding), weighted statistics were computed by converting the total sum scores (range 0–28) back to the 0–2 item metric. The weighted mean was M = 1.30 (SD = 0.28), indicating that responses fell above the egalitarian midpoint (i.e., leaning towards stereotypical adherence). Males (M = 1.42, SD = 0.28) reported significantly higher adherence to stereotypical gender roles than females (M = 1.19, SD = 0.22).

For the GTI (range: 0–2, with 0 = egalitarian, 1 = counter-stereotypical and 2 = stereotypical responding), the weighted mean was M = 0.89 (SD = 0.47), slightly below the midpoint. Gender differences were minimal (females: M = 0.87; males: M = 0.92), consistent with the negligible d = 0.11 effect size.

Item-level response patterns are reported in Supplementary Tables S78–S80. GRAS and GTI item-level response frequencies by gender and school type are provided in Supplementary Tables S85–S86 and S88.

#### 3.6.2. Percentile Distributions

Percentile distributions for sum scores are provided in Supplementary Table S71. For the GSAS total (sum range: 17–68), the weighted median was 32 (IQR: 26–39). Gender-stratified percentiles revealed a substantial separation in distributions: the median score for males (37) approached the 90th percentile for females (38).

#### 3.6.3. Operational Cutoffs

Two complementary approaches were used to establish operational cutoffs for identifying elevated stereotype endorsement.

Percentile-based cutoffs (Supplementary Table S72) were established at P75 (Elevated) and P90 (Alert) thresholds. For the GSAS total, the Elevated cutoff (≥38) identified 30.1% of the weighted sample (11.1% of females, 49.2% of males), while the Alert cutoff (≥43) identified 15.7% (3.6% of females, 27.9% of males).

Kernel density estimation (KDE) natural breaks (Supplementary Table S73) identified empirical score groupings based on density antimodes. For the GSAS total, natural breaks occurred at 26 and 36 (sum scores), corresponding to the boundaries between Low, Medium, and Elevated categories. Hartigan’s dip test confirmed a significant departure from unimodality (*p* < 0.001), supporting the appropriateness of categorical classification.

A combined classification system integrating percentile-based and KDE-based approaches was developed for the GSAS (Supplementary Table S74). Five categories were established: Low (sum 17–25; 23.1%), Low-Medium (sum 26–35; 37.6%), High-Medium (sum 36–37; 6.7%), Elevated (sum 38–42; 15.3%), and Very Elevated (sum ≥43; 17.3%). The gender disparity in categorical distributions was substantial: in the Low category, females outnumbered males nearly 4:1 (39.5% vs. 10.5%), whereas in the Very Elevated category, males outnumbered females approximately 8:1 (27.9% vs. 3.6%). This pattern underscores that the same absolute score carries different normative implications depending on gender (see Figure 2). Descriptive statistics by classification category and cross-tabulation of natural breaks with percentile categories are reported in Supplementary Tables S76 and S77.

Item-level response frequencies and norms are provided in Supplementary Tables S78–S80. Full administration forms and scoring algorithms are documented in Supplementary Materials Files S10–S13.

## 4. Discussion

The present study developed and validated the Gender Stereotypes and Roles Adherence Battery for Adolescents (GAB-A), a multi-module assessment tool for measuring endorsement of gender stereotypes among Italian adolescents. Comprehensive psychometric evaluation across content validity, factorial structure, reliability, known-groups validity, convergent and discriminant validity, and measurement invariance demonstrates that the GAB-A provides a psychometrically sound instrument for research and applied contexts. Beyond confirming the technical adequacy of the scales, the validation process revealed several theoretically significant findings regarding the structure, correlates, and demographic patterns of gender stereotype endorsement that warrant detailed discussion.

### 4.1. Summary of Psychometric Properties

The GAB-A demonstrated strong psychometric properties across all three modules. Content validity assessment by a multidisciplinary expert panel (N = 12) yielded excellent scale-level indices (S-CVI/Ave = 0.967), with 97.8% of items meeting the I-CVI ≥ 0.78 threshold for acceptability. The split-sample cross-validation strategy—with EFA conducted on Sample A (n = 1479) and CFA on the independent Sample B (n = 1476)—provided robust evidence for the hypothesized factorial structures while protecting against overfitting.

The GSAS (Gender Stereotyped Attitude Scale) emerged as a 17-item measure with three correlated factors: Traditional Stereotypes (GSAS-TS; 9 items), Violence and Sexuality Myths (GSAS-VM; 5 items), and Relational Control (GSAS-RC; 3 items). CFA demonstrated good-to-excellent fit (CFI = 0.950, TLI = 0.941, RMSEA = 0.049, SRMR = 0.037), and internal consistency was excellent (α = 0.894). This three-factor structure aligns with theoretical distinctions in the gender literature between traditional role beliefs, hostile attitudes toward women, and controlling relationship behaviors (Glick & Fiske, 1996). The GRAS (Gender Role Activities Scale) demonstrated a two-factor structure distinguishing Leisure Activities (GRAS-LA; 5 items) from Social Roles (GRAS-SR; 9 items), with excellent fit (CFI = 0.989, TLI = 0.987, RMSEA = 0.055, SRMR = 0.049) and good reliability (α = 0.850). The GTI (Gendered Traits Inventory) showed a unidimensional structure with acceptable fit (CFI = 0.948, RMSEA = 0.075) and good reliability (α = 0.802). All scales achieved full or partial scalar measurement invariance across gender and school type, supporting valid group comparisons.

### 4.2. The Three-Module Structure: Related but Distinct Constructs

Inter-scale correlations revealed that the three GAB-A modules capture related but distinguishable facets of gender stereotype endorsement. The GSAS and GRAS showed a strong correlation (r = 0.653), indicating substantial shared variance (43%) while maintaining sufficient uniqueness (57%) to justify separate assessment. The GTI correlated more modestly with both the GSAS (r = 0.312) and GRAS (r = 0.440). This correlational pattern supports a hierarchical conceptualization wherein attitudinal and role-based stereotypes form a closely related cluster reflecting prescriptive beliefs about what men and women should do, whereas trait stereotypes represent more fundamental descriptive beliefs about what men and women are (Eagly & Wood, 2012). A recent systematic review of the ambivalent sexism literature (Bareket & Fiske, 2023) reinforces this distinction, demonstrating that hostile sexism functions primarily to protect men’s power, whereas benevolent sexism serves to guard traditional gender role arrangements. This pattern is consistent with the differentiation between the GSAS-VM (which captures hostile attitudes) and GSAS-TS (which reflects benevolent sexism and beliefs about complementary roles).

The stronger GTI–GRAS association (compared to GTI–GSAS) may reflect conceptual overlap between attributing personality traits to genders and believing genders are suited for corresponding activities, as both tap into essentialist reasoning about “natural” gender differences (Haslam et al., 2000). From a social role theory perspective (Eagly & Wood, 2012), individuals who endorse role-based stereotypes may be particularly likely to invoke essentialist trait explanations to justify those role assignments.

Within the GSAS, the three subscales showed substantial but not redundant intercorrelations (rs = 0.47–0.68), supporting the utility of subscale-level assessment. The GSAS-RC (Relational Control) subscale emerged as particularly noteworthy given its distinct pattern of external correlates. Although strongly correlated with the other GSAS factors, GSAS-RC showed the strongest association with physical aggression (r = 0.24) while demonstrating consistent positive associations with distress across both gender groups, a pattern not observed for the other subscales. This suggests that beliefs legitimizing partner surveillance may represent a particularly problematic dimension of gender ideology with unique implications for relationship behavior and psychological functioning. Furthermore, its unique link to distress suggests that beliefs legitimizing partner surveillance may not be purely ideological, but also functionally related to relational insecurity. It is plausible that distress acts as an antecedent or mediator, wherein individuals with higher anxiety endorse controlling beliefs as a defensive strategy. Thus, GSAS-RC may capture a dimension of gender ideology that is particularly intertwined with the individual’s psychological state, serving as a cognitive scaffold for underlying insecurity.

### 4.3. Gender Differences Across Scales: Theoretical Implications

The known-groups validity analyses revealed dramatically different patterns of gender differences across the three GAB-A modules, with important implications for theories of gender stereotype development and maintenance.

#### 4.3.1. Large Gender Differences on Attitudinal and Role-Based Measures

For the GSAS and GRAS, males showed substantially higher scores than females, with effect sizes in the large range (GSAS: d = 1.07, 95% CI [0.99, 1.15]; GRAS: d = 0.88, 95% CI [0.81, 0.96], dlatent = 1.06). These findings replicate and extend prior research documenting that males more strongly endorse traditional gender stereotypes across diverse samples and measures (Glick et al., 2000; Swim et al., 1995). The pattern is consistent with recent evidence from the Italian adult population showing persistence of gender stereotypes regarding occupations and traits despite high educational attainment (Carvalho Silva et al., 2024), and with findings from Spanish adolescents linking egalitarian attitudes to lower internalization of both hostile and benevolent sexism (Bonilla-Algovia et al., 2024). From a system justification perspective (Jost & Banaji, 1994), male endorsement of gender stereotypes may serve to legitimize existing social arrangements that disproportionately benefit men in terms of economic resources, political power, and social status.

The consistency of gender differences across GSAS subscales is particularly informative. The largest effect emerged for Violence and Sexuality Myths (GSAS-VM; d = 1.04), suggesting that male adolescents in this sample are substantially more likely than female adolescents to endorse beliefs that normalize sexual coercion and intimate partner violence. This finding aligns with research linking masculine gender ideology to tolerance of relationship aggression (Parrott & Zeichner, 2003) and highlights the potential relevance of stereotype reduction interventions for violence prevention.

#### 4.3.2. Negligible Aggregate Differences Masking Strategic Self-Serving Biases

In stark contrast to the attitudinal measures, the aggregate gender difference on the Trait Inventory (GTI) appeared negligible (d = 0.11, 95% CI [0.04, 0.18]). On a macroscopic level, both male and female adolescents endorsed the same trait stereotypes at similar rates, that males are more aggressive and self-confident, while females are more sensitive and cooperative. This surface-level uniformity suggests that essentialist beliefs about gendered personality are deeply internalized cultural schemas that transcend personal gender identity (Eagly & Steffen, 1984). In this case, respondents fail to recognize the socially constructed nature of the phenomenon, instead misinterpreting it as a personality trait. However, a granular analysis of the Self-Attribution Gap (Δ), defined as the difference between a group’s self-rating and the outgroup’s rating of them (Supplementary Table S87), reveals that this consensus is actively negotiated to preserve positive social identity. Using Bonferroni-corrected z-tests (αadj < 0.0025) to assess the 20 target-item combinations, significant self-serving biases were identified that challenge the assumption of passive internalization.

The analysis revealed two distinct forms of identity negotiation. First, absolute ingroup bias (Directional Discordance). For the highly valued trait of Independence, the groups disagreed on the direction of the stereotype. Both males and females significantly claimed the trait for themselves (males, Self: 32.5% vs. Other: 12.6%; females, Self: 20.2% vs. Other: 8.8%). This “double self-serving bias” (ΔM = +19.9%; ΔF = +11.4%, *p* < 0.001) indicates that for traits central to agency and autonomy, both genders reject outgroup dominance to maintain collective self-esteem.

Second, Relative Ingroup Bias (Intensity Discordance): For traits where the direction was agreed upon, the in-group consistently optimized the magnitude. Enhancement: While both groups agreed females are more Reasonable and Cooperative, female respondents endorsed these positive traits at significantly higher rates than males acknowledged (Δ between 9% and 14%, *p* < 0.001). Deflection: Conversely, for negative traits like Fragility, females engaged in defensive deflection, endorsing the trait significantly less (48.5%) than males attributed it to them (54.6%; Δ = −6.1%, *p* = 0.019).

Interestingly, males exhibited a distinct pattern regarding Unpredictability. While females attempted to deflect this negative trait (Δ = −8.0%), males admitted to it significantly more often than females accused them of it (Self: 21.5% vs. Other: 17.0%; *p* < 0.05). This divergence suggests the operation of a gendered double code, wherein females likely reject the term as a marker of emotional instability, whereas males embrace it as a proxy for agency and strategic autonomy.

To test for gender differences in bias strategies, a standardized Self-Serving Score (SSS) was computed for each item. A distinct asymmetry was found in bias expression: Males exhibited a dominant strategy of self-enhancement (Mean SSSpos = 11.0% vs. SSSneg = −1.2%), significantly inflating their association with positive traits. In contrast, Females utilized a dual strategy, showing moderate enhancement (7.9%) alongside significant deflection (4.9%).

These findings reframe the theoretical interpretation of the GTI. While the low Cohen’s d indicates shared access to cultural stereotypes, the item-level data suggest these schemas are not accepted passively. Instead, adolescents engage in a dynamic process of Social Identity negotiation (Tajfel & Turner, 1979), accepting the broad cultural script but statistically manipulating specific attribution probabilities to maximize positive distinctiveness. The GTI thus taps into a hybrid construct: the knowledge of descriptive norms (“how men and women are”) filtered through the protective lens of in-group favoritism.

The successful implementation of Schema B scoring for the GTI reinforces this interpretation of trait attribution as an active, identity-relevant process. Under Schema B, the egalitarian response (“It doesn’t matter”) is coded as 0, representing a refusal to engage in gender categorization. Crucially, counter-stereotypical responses are coded as 1, grouping them with traditional stereotypes rather than treating them as “opposites.”

This scoring structure aligns with our finding that both stereotype endorsement and self-serving reversals are manifestations of the same underlying psychological mechanism: gender salience. Whether an adolescent adheres to the traditional script or flips it to favor their in-group, they are engaged in gendered categorization. The dramatic improvement in reliability under Schema B (α improved from 0.515 to 0.802) confirms that the fundamental construct being measured is not a linear continuum from “Sexist” to “Progressive,” but a categorical distinction between those who use gender as a primary explanatory lens (for tradition or self-enhancement) and those for whom gender is irrelevant to personality attribution.

### 4.4. Empirical Verification of Stereotype Directions: Cultural Change in Gender Beliefs?

Evidence of cultural evolution in gender stereotypes emerged across multiple GAB-A scales, necessitating empirical verification of item characteristics rather than reliance on classical theoretical assumptions.

#### 4.4.1. Stereotype Direction Recoding in the GTI

The empirical verification of stereotype directions for the GTI revealed that two items required coding opposite to classical theoretical expectations. Unpredictability, traditionally associated with feminine emotionality (Williams & Best, 1990), was empirically associated with males in the present sample (19.5% attributed to males vs. 14.7% to females). Reasonableness, classically associated with masculine rationality, was empirically associated with females (27.0% vs. 10.2%).

These findings may reflect cultural evolution in gender stereotypes among contemporary Italian adolescents. The association of unpredictability with males, rather than the historically stereotyped “emotional female,” may reflect contemporary framings of masculinity that emphasize impulsivity, risk-taking, and poor emotional regulation (Levant et al., 2009). This interpretation is strongly supported by our bias analysis: while females actively deflected this trait (ΔF = −8.0%), males engaged in a unique pattern of “acceptance,” attributing it to themselves significantly more often than females did (ΔM = +4.5%, *p* = 0.044), effectively validating it as a trait that male adolescents may paradoxically embrace as markers of agency rather than instability.

Similarly, the association of reasonableness with females may reflect benevolent sexism beliefs (Glick & Fiske, 1996) that characterize women as more mature, responsible, and sensible than men; on the other hand, it could be an active reclamation of rationality by young women. Indeed, female respondents exhibited their strongest self-enhancement effect on this item (ΔF = +14.1%, *p* < 0.001), suggesting this shift is not just an imposed restriction to caretaking roles, but also a strategic rejection of historical narratives regarding female instability.

When items 7 and 10 were coded according to theoretical rather than empirical directions, reliability dropped to unacceptable levels (α = 0.359), demonstrating that coding decisions can dramatically affect scale psychometric properties.

#### 4.4.2. Item Exclusions Reflecting Evolving Stereotypes in the GRAS

Similar evidence of cultural change emerged during GRAS item analysis, where two items were excluded partly because their gender associations appeared to be evolving (Supplementary Table S84). The item “Talking on the phone” (rit = 0.29) showed weak psychometric properties. What was once a stereotypically feminine activity (extended phone conversations) has become gender-neutral in an era when smartphone use is universal among adolescents. Similarly, “Teaching” (rit = 0.28) was excluded partly because it was “perceived as gender-neutral” by contemporary respondents, despite teaching historically being stereotyped as a feminine profession in many cultural contexts.

A third excluded item, “Making scientific discoveries” (rit = 0.24), showed the lowest item-total correlation in the GRAS. The low discrimination may reflect the abstract nature of the concept, but it could also indicate increased egalitarian attitudes toward women in science among contemporary adolescents, a possibility consistent with educational initiatives promoting STEM participation among girls.

#### 4.4.3. Implications for Scale Development

These patterns across both the GTI and GRAS underscore the necessity of empirical verification before applying theoretical assumptions from classic cross-cultural research to contemporary adolescent populations. Gender stereotypes are not static cultural artifacts but evolving belief systems that respond to technological change (smartphone ubiquity), educational initiatives (women in STEM), and shifting cultural narratives about masculinity and femininity. Importantly, such evolution is not necessarily linear toward egalitarianism; recent research has documented patterns of “retrenchment” or countertrends among younger cohorts, suggesting that stereotype change may be nonmonotonic and context-dependent (Palomino-Suárez & Aparicio García, 2025). Scale developers working with gender stereotype content should routinely verify stereotype directions and item functioning empirically rather than assuming that classical theoretical expectations will hold in new populations or time periods.

### 4.5. Stereotype–Wellbeing Relationships: Gender Moderation and Simpson’s Paradox

The criterion validity analyses revealed complex, gender-moderated relationships between stereotype endorsement and psychological wellbeing that require careful interpretation.

#### 4.5.1. Aggregate Correlations: A Misleading Pattern

Aggregate correlations between GSAS/GRAS scores and psychological wellbeing measures initially showed an unexpected pattern: negative associations with distress (GSAS: r = −0.149; GRAS: r = −0.184), hostility, and alexithymia, alongside positive associations with self-esteem. If taken at face value, these correlations would suggest a “protective” effect of stereotype endorsement, suggesting that holding traditional gender beliefs is associated with better psychological outcomes.

#### 4.5.2. Within-Group Patterns: Reversing the Aggregate Effect

Gender-stratified analyses revealed that the aggregate pattern was a statistical artifact produced by confounding between gender, stereotype endorsement, and wellbeing outcomes. Among males, stereotype endorsement showed positive associations with distress (GSAS: r = 0.066, *p* = 0.007), hostility (r = 0.085, *p* < 0.001), and alexithymia (r = 0.095, *p* < 0.001), and a negative association with self-esteem (r = −0.075, *p* = 0.002). Among females, these associations were non-significant or weakly negative.

**Figure 3:**
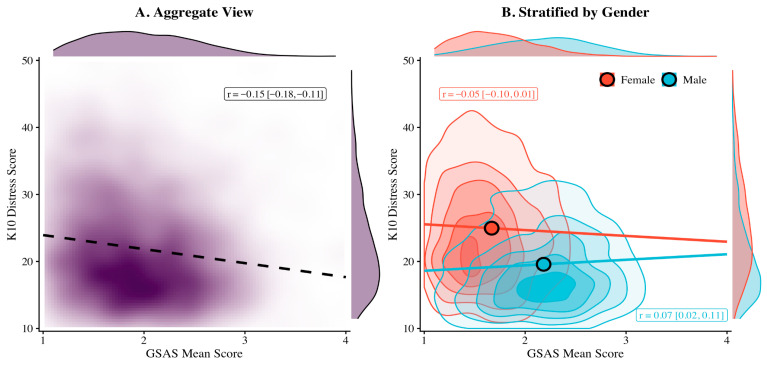
Simpson-type aggregation effect in the association between gender-stereotype endorsement and psychological distress. Note. GSAS = Gender Stereotyped Attitude Scale (higher scores indicate greater endorsement). K10 = Kessler Psychological Distress Scale (higher scores indicate greater distress). Panel (**A**) shows the aggregate association; Panel (**B**) shows associations stratified by gender. Shaded regions and contour lines represent the bivariate distribution of observations (kernel density estimate); marginal density plots are displayed along the top (GSAS) and right (K10). Solid and dashed lines represent fitted linear trends. Points indicate group means (centroids). Pearson correlations (r) are reported with 95% confidence intervals in brackets. Results illustrate that the negative pooled association does not reflect the within-gender patterns, consistent with a Simpson-type aggregation effect (aggregation bias).

This pattern constitutes a textbook example of Simpson’s paradox (Kievit et al., 2013), wherein aggregate correlations can be misleading when subgroup differences exist. Notably, this statistical phenomenon has recently been documented in the gender equality literature more broadly, where country-level associations between gender equality and outcomes such as occupational segregation reverse direction when individual-level variation is considered (Berggren & Bergh, 2025). The “protective” aggregate correlation arose because: (a) males had substantially higher stereotype endorsement than females, (b) males and females differed on wellbeing variables, and (c) within each gender group, the relationship between stereotypes and wellbeing was null (females) or in the theoretically expected positive direction (males). The aggregation across groups created a spurious negative correlation driven by between-group rather than within-group variation (see Figure 3).

#### 4.5.3. Theoretical Implications

The gender-moderated pattern has important theoretical implications. For male adolescents, endorsement of traditional gender stereotypes was associated with poorer psychological outcomes, specifically higher distress, hostility, and alexithymia, and lower self-esteem. This finding is consistent with research on the costs of traditional masculinity ideology, which has been linked to restricted emotionality, reluctance to seek help, greater self-stigma regarding psychological services, and both internalizing and externalizing problems (Wong et al., 2017; Üzümçeker, 2025).

For female adolescents, stereotype endorsement showed essentially no relationship with wellbeing outcomes. This null pattern may reflect the complex role of benevolent sexism for women, which can simultaneously offer subjective benefits (e.g., feeling protected and cherished) while functioning to maintain inequality (Glick & Fiske, 2001). Alternatively, the low variance in female stereotype endorsement (substantially lower means with compressed distributions) may have attenuated correlations. Future research should examine whether the null pattern holds across samples with greater variability in female stereotype endorsement.

Two exceptions to the general pattern merit emphasis. First, the GSAS-RC subscale (Relational Control) showed consistent positive associations with distress in both genders (males: r = 0.083, *p* < 0.001; females: r = 0.062, *p* = 0.026). Beliefs legitimizing partner surveillance appear to be uniquely associated with distress regardless of gender, perhaps because such beliefs implicate interpersonal mistrust and control dynamics that are inherently stressful. Second, the GTI showed positive correlations with distress in both subgroups (males: r = 0.110, *p* < 0.001; females: r = 0.087, *p* = 0.002). Essentialist trait attributions, unlike prescriptive role beliefs, appear to be uniformly associated with poorer psychological outcomes across genders. This may be due to the mediating role of psychological rigidity, intolerance of uncertainty and need for cognitive closure (Roets et al., 2015). Individuals with a rigid cognitive style often experience higher distress due to an intolerance of ambiguity and simultaneously endorse essentialist stereotypes to impose structure and certainty on their social world (Allport, 1954; Bastian & Haslam, 2006).

The methodological lesson of this analysis extends beyond the present study. Researchers investigating relationships between stereotype endorsement and psychological outcomes should routinely test for demographic confounding and moderation. Failure to do so risks drawing spurious conclusions from aggregate analyses.

### 4.6. Measurement Invariance: Implications for Group Comparisons

Measurement invariance testing represents a critical prerequisite for valid group comparisons (Chen, 2007; Putnick & Bornstein, 2016). The present findings have important implications for how the GAB-A should be used in research and practice.

#### 4.6.1. Full Scalar Invariance Across School Type

All three GAB-A scales achieved full scalar invariance across the three school types (Academic, Technical, Vocational). This finding indicates that the scales measure the same constructs in the same way across educational tracks, with equivalent factor loadings and item thresholds.

The school type differences observed in the present study, though smaller than gender differences (GSAS: η^2^ = 0.064; GRAS: η^2^ = 0.021; GTI: η^2^ = 0.003), have practical significance for targeting interventions. Technical and vocational students showed the highest stereotype endorsement on most scales, suggesting that educational context (including social background, curriculum, peer culture, and occupational expectations associated with different tracks) may shape gender-related attitudes. The documented invariance supports valid identification of elevated endorsement profiles for targeted prevention programs.

#### 4.6.2. Scalar Invariance Across Gender: Scale-Specific Patterns

The measurement invariance findings across gender were more complex and scale-specific. The GSAS achieved full scalar invariance with all 17 items, indicating that the scale functions equivalently for male and female adolescents. This is a particularly robust finding given the large gender differences in mean levels; it indicates that these mean differences reflect genuine attitudinal differences rather than differential item functioning.

The GRAS similarly achieved full scalar invariance after removal of one item (Driving) that showed substantial differential item functioning (female λ = 0.39 vs. male λ = 0.70). This DIF pattern is substantively interesting: driving appears to be more central to males’ conception of gendered activities than to females’. The removal of this item, already excluded based on low item-total correlations, ensured valid measurement across gender groups.

The GTI achieved partial scalar invariance, with 8 of 10 items (80%) functioning equivalently across gender groups. Two items (Independence and Reasonableness) showed threshold non-invariance. However, considering our item-level bias analysis, this non-equivalence appears to stem not from differing semantic interpretations, but from competing self-enhancement strategies. These two items elicited the strongest self-serving biases in the entire scale: Independence showed the largest male enhancement (Δ = +19.9%) and the second largest female enhancement (Δ = +11.4%), while Reasonableness showed the largest female enhancement (Δ = +14.1%) alongside the second largest male enhancement (Δ = +12.0%). The lack of scalar invariance here likely captures the intensity of this identity negotiation, where both groups aggressively claim these high-value traits, rather than a failure of measurement validity. Given that 80% of items retained scalar constraints, comparison of latent means remains defensible (Putnick & Bornstein, 2016), though researchers should interpret gender comparisons on these specific traits as reflective of both stereotype content and active identity management.

### 4.7. Comparison with Existing Measures

The GAB-A addresses several gaps in the existing measurement landscape for gender stereotype assessment among adolescents.

#### 4.7.1. Relation to Ambivalent Sexism Measures

The Ambivalent Sexism Inventory (ASI; Glick & Fiske, 1996) remains the most widely used measure of sexist attitudes, distinguishing hostile sexism (antipathy toward women who violate traditional gender norms) from benevolent sexism (subjectively positive but patronizing attitudes toward women). The GSAS shows conceptual overlap with the ASI, particularly in the GSAS-TS subscale, which captures beliefs about complementary gender roles similar to the ASI’s benevolent sexism subscale.

However, the GAB-A extends beyond the ASI in several ways. First, the GSAS-VM subscale specifically targets beliefs about gender-based violence and sexual coercion, content that is not systematically assessed by the ASI but is critically important for adolescent populations given developmental considerations around dating violence and consent. Second, the GSAS-RC subscale assesses partner surveillance beliefs that have gained relevance in the digital age (e.g., social media password access, location tracking). Third, the GRAS and GTI provide a systematic assessment of activity and trait stereotypes, which are not captured by purely attitudinal measures.

#### 4.7.2. Adolescent-Appropriate Content

Many existing stereotype measures were developed and validated with adult samples, raising questions about developmental appropriateness and cultural relevance for adolescents. The GAB-A was developed specifically for Italian adolescents, with items reflecting developmentally relevant domains (e.g., football/soccer as a stereotypically male sport in Italian culture, academic subject aptitudes, leisure activities). The normative data and cutoffs established in the present study provide age-appropriate reference points that adult-normed measures cannot offer.

#### 4.7.3. Multi-Module Assessment

The modular structure of the GAB-A, assessing attitudes (GSAS), role beliefs (GRAS), and trait attributions (GTI) separately, provides more differentiated assessment than single-dimension measures. The differential pattern of findings across modules (e.g., large vs. negligible gender differences; different correlational patterns with wellbeing) demonstrates the value of this multi-faceted approach. Researchers and practitioners can select modules relevant to their specific assessment goals while maintaining psychometric integrity.

### 4.8. Practical Applications

#### 4.8.1. Identifying Elevated Endorsement Profiles

The normative data and operational cutoffs established in this study enable practical use of the GAB-A for identifying elevated endorsement profiles. The combined classification system for the GSAS identifies approximately 27% of the total sample as having “Elevated” or “Very Elevated” stereotype endorsement (sum score ≥ 39). This proportion rises to 45% among males and drops to 8% among females, highlighting the importance of gender-stratified interpretation.

The GSAS-VM (Violence and Sexuality Myths) and GSAS-RC (Relational Control) subscales may be particularly valuable for screening in contexts concerned with relationship violence prevention. Elevated scores on these subscales showed the strongest associations with physical aggression and were associated with distress across both genders. The relevance of such screening is supported by recent evidence linking sexism to teen dating violence, with gender-differentiated mediation pathways involving personal distress in males and assertiveness deficits in females (Villanueva-Blasco et al., 2024). School-based prevention programs targeting dating violence might use these subscales to identify elevated endorsement profiles for more intensive intervention.

#### 4.8.2. Intervention Targeting

The school type differences documented in this study, combined with the demonstrated measurement invariance across educational tracks, support the use of GAB-A for targeting interventions by educational context. Technical and vocational students showed consistently higher stereotype endorsement than academic students, suggesting that prevention programs might productively focus resources on technical and vocational educational settings. The additive (non-interactive) pattern of gender and school type effects indicates that male students in the non-academic track represent a particularly vulnerable group.

#### 4.8.3. Monitoring Intervention Effects

The established reliability and measurement invariance properties of the GAB-A support its use for monitoring change over time. The demonstrated stability of factor structures across split samples and demographic subgroups suggests that pre-post differences would reflect genuine attitude change rather than measurement artifacts. Researchers evaluating gender equality interventions should consider the GAB-A as an outcome measure, particularly given the availability of multiple construct-specific subscales that can detect targeted changes.

### 4.9. Limitations

Several limitations warrant consideration when interpreting the present findings.

#### 4.9.1. Sample Characteristics

The validation sample was limited to Italian adolescents from the Province of Rome, raising questions about generalizability to other Italian regions, age groups, or cultural contexts. The overrepresentation of males (56.4%) reflects differential enrollment patterns across Italian secondary education tracks rather than sampling bias. Future validation efforts should include samples from other Italian regions, different age groups, and cross-cultural samples to establish broader generalizability.

#### 4.9.2. Cross-Sectional Design

The present validation is based on cross-sectional data from the first wave of data collection, which precludes causal inference regarding the observed associations between stereotype endorsement and psychological outcomes. Although stereotype endorsement was observed to be associated with distress among males, it cannot be determined whether stereotype beliefs cause psychological difficulties, whether psychological difficulties lead to stereotype adoption, or whether third variables (e.g., family context, media exposure) influence both. However, the present study is part of the MIB (Mutamenti Interazionali e Benessere) longitudinal research project, which will follow participating students across all five years of Italian secondary school. Future waves of data collection will enable testing of temporal precedence, developmental trajectories of stereotype endorsement, and potential bidirectional effects between stereotypes and psychological outcomes.

#### 4.9.3. Self-Report Methodology

All measures relied on self-report, introducing potential shared method variance and social desirability bias. Although response style analyses indicated minimal impact of acquiescence and extreme responding on scale scores, social desirability effects cannot be ruled out, particularly for overtly prejudicial items in the GSAS-VM subscale. Future research might incorporate implicit measures or behavioral assessments to complement self-report data.

#### 4.9.4. Measurement Invariance Limitations

Although the present study established measurement invariance across gender and school type, invariance testing for the GTI was limited by convergence issues with the sparse categorical data, resulting in partial rather than full scalar invariance. The two non-invariant items (Independence, Reasonableness) may be interpreted somewhat differently by males and females. Researchers using the GTI for gender comparisons should acknowledge this limitation.

#### 4.9.5. Criterion Measure Selection

The criterion measures used for convergent and discriminant validity assessment (BPAQ, K10, RSES, PAQ-S) represent a limited sampling of relevant constructs. Future validation efforts should examine associations with additional theoretically relevant variables, including relationship quality, dating behavior, academic/occupational aspirations, and behavioral measures of discrimination and prejudice. In addition, the percentile-based thresholds proposed in the present study are distribution-based and should be interpreted as normative or operational categories rather than risk cutoffs.

#### 4.9.6. Cluster Sampling Design

Although the cluster sampling design (students nested within schools) may introduce non-independence of observations, several design features mitigate this concern: the two-dimensional stratification of school selection (by educational track and geographical area) reduces systematic between-school variability, the inclusion of only first-year students limits school-level socialization effects, and the standardized CAPI administration protocol minimizes context effects. Nevertheless, formal multilevel modeling with school-level identifiers would allow precise partitioning of variance components, and future waves of the longitudinal study should incorporate this approach.

#### 4.9.7. Cognitive Pretesting

Although a formal cognitive pretesting study (e.g., think-aloud protocols) was not conducted, the iterative refinement process—grounded in a decade of direct engagement with the target population—provides strong ecological validity for item content and wording. Future instrument development should include formal cognitive testing.

### 4.10. Future Directions

Several directions for future research emerge from the present findings.

#### 4.10.1. Longitudinal and Developmental Research

Longitudinal designs could establish whether stereotype endorsement predicts psychological outcomes over time and how stereotypes develop and change across adolescence. The negligible gender differences observed for trait stereotypes (GTI) raise questions about whether this pattern is present earlier in development or emerges through adolescence. Tracking stereotype trajectories from middle school through late adolescence would illuminate developmental processes.

#### 4.10.2. Cross-Cultural Validation

The GAB-A was developed and validated in an Italian context, and its applicability to other cultural settings requires empirical evaluation. Some content (e.g., football as stereotypically male) may be culture-specific, while other content (e.g., beliefs about domestic roles) may show cross-cultural invariance. Cross-cultural validation studies, particularly in Southern European and Mediterranean contexts with similar gender role traditions, would enhance the international utility of the battery.

#### 4.10.3. Intervention Research

The present findings provide a foundation for intervention research. Experimental studies could test whether evidence-based educational programs produce measurable reductions in GAB-A scores and whether such reductions mediate improvements in relationship quality or reductions in aggressive behavior. The demonstrated measurement invariance supports valid pre-post comparisons within treatment and control groups.

#### 4.10.4. Extension to Gender-Diverse Populations

The present validation focused on binary gender comparisons (male vs. female), excluding 20 participants who identified as non-binary or did not report gender. Future research should explicitly include and validate the GAB-A with gender-diverse populations. Theoretical frameworks on gender stereotypes have been critiqued for implicitly assuming binary gender categories (Hyde et al., 2019), and empirical extension to non-binary individuals represents an important direction. Leaper (2024) has recently proposed an expanded developmental model of ambivalent sexism that addresses gender-diverse youth and emphasizes cultural variation in stereotype content, providing both theoretical grounding and methodological rationale for such validation efforts.

#### 4.10.5. Integration with Implicit Measures

The present study relied exclusively on explicit, self-report measures. Integration with implicit measures (e.g., Implicit Association Test) would enable examination of whether explicit and implicit stereotype measures show convergent or divergent patterns and whether implicit measures add predictive validity for behavioral outcomes beyond explicit measures.

## 5. Conclusions

The Gender Stereotypes and Roles Adherence Battery for Adolescents (GAB-A) provides a psychometrically sound, multi-dimensional assessment tool for measuring gender stereotype endorsement among Italian adolescents. The three modules (GSAS, GRAS, and GTI) capture related but distinct facets of gender-related beliefs, with trait stereotypes (GTI) emerging as a qualitatively different construct characterized by near-uniform endorsement across gender groups.

The relationship between stereotype endorsement and psychological wellbeing proved to be moderated by gender, with important methodological implications. What initially appeared to be a paradoxical “protective” pattern was revealed through stratified analyses to be a Simpson’s paradox artifact. Among male adolescents, stereotype endorsement showed the theoretically expected positive association with psychological distress, while among females, no significant association emerged. These findings underscore the importance of testing for demographic confounding and moderation in stereotype research.

The documented measurement invariance across gender and school type supports valid group comparisons, enabling both research applications (testing theoretical predictions about group differences) and practical applications (identifying elevated endorsement profiles for interventions). The present findings provide strong initial evidence of structural validity, internal consistency, and measurement invariance, together with gender-stratified normative data. The interdisciplinary nature of the study, which enabled the validation of the GAB-A, also enriched the analysis by incorporating findings aimed at elucidating the key factors underlying the generational reproduction of gender stereotypes. Furthermore, the extensive psychometric component of the Interactional Changes and Wellbeing (MIB) project questionnaire revealed several noteworthy associations with adolescents’ psychological well-being; however, at this stage of the project, causal relationships cannot yet be established. For researchers, the GAB-A offers a validated instrument for investigating gender stereotype development and consequences in Italian adolescent populations. For practitioners, it provides a tool with normative data and empirically derived cutoffs for identifying elevated stereotype endorsement that may warrant intervention.

## Figures and Tables

**Table 1:** Sample Characteristics (N = 2955).

Characteristic	Total (N = 2955)	Female (n = 1289)	Male (n = 1666)	Test Statistic
Age, M (SD)	14.30 (0.56)	14.29 (0.56)	14.31 (0.56)	-
School Type, n (%)				χ^2^(2) = 56.27 ***
Academic	1390 (47.0)	675 (52.4)	715 (42.9)	
Technical	989 (33.5)	336 (26.1)	653 (39.2)	
Vocational	576 (19.5)	278 (21.6)	298 (17.9)	
Citizenship, n (%)				χ^2^(1) = 6.72 *
Italian	2656 (89.9)	1137 (88.2)	1519 (91.2)	
Foreign	299 (10.1)	152 (11.8)	147 (8.8)	
Only Child, n (%)				χ^2^(1) = 0.64
Yes	543 (18.4)	228 (17.7)	315 (18.9)	
No	2412 (81.6)	1061 (82.3)	1351 (81.1)	
Parental Education, n (%)				χ^2^(3) = 5.94
Low	699 (23.7)	321 (24.9)	378 (22.7)	
Medium	986 (33.4)	443 (34.4)	543 (32.7)	
Medium-High	526 (17.8)	227 (17.6)	299 (18.0)	
High	738 (25.0)	296 (23.0)	442 (26.6)	
Missing	6 (0.2)	2 (0.2)	4 (0.2)	
Household Income, n (%)				χ^2^(2) = 5.48
Low	446 (15.1)	215 (16.7)	231 (13.9)	
Medium	1481 (50.1)	622 (48.4)	859 (51.8)	
High	1014 (34.3)	447 (34.8)	567 (34.2)	
Missing	14 (0.5)	5 (0.4)	9 (0.5)	
Academic Performance (Self-Reported), n (%)				χ^2^(2) = 2.07
High	632 (21.4)	275 (21.3)	357 (21.4)	
Medium	2024 (68.5)	895 (69.4)	1129 (67.8)	
Low	299 (10.1)	119 (9.2)	180 (10.8)	

Note. Parental education = combined highest educational attainment across both parents. * *p* < 0.05. *** *p* < 0.001.

**Table 2:** Split-Sample Equivalence Testing.

Variable	Sample A (n = 1479)	Sample B (n = 1476)	Test Statistic	*p*
Gender (% female)	43.6%	43.6%	χ^2^ < 0.001	>0.999
Age, M (SD)	14.32 (0.56)	14.28 (0.55)	t = 1.85	0.064
School Type			χ^2^ = 0.001	0.999
Academic	47.1%	47.0%		
Technical	33.5%	33.5%		
Vocational	19.5%	19.5%		
Citizenship (% Italian)	89.3%	90.5%	χ^2^ = 1.17	0.280
Only Child (% yes)	18.6%	18.2%	χ^2^ = 0.07	0.796
Parental Education			χ^2^ = 3.72	0.293
Low	23.1%	24.3%		
Medium	33.4%	33.4%		
Medium-High	17.1%	18.6%		
High	26.4%	23.6%		
Household Income			χ^2^ = 0.86	0.650
Low	14.8%	15.6%		
Medium	51.2%	49.5%		
High	34.0%	34.9%		
Academic Performance			χ^2^ = 0.91	0.635
High	21.0%	21.8%		
Medium	68.4%	68.6%		
Low	10.6%	9.6%		

Note. Sample A used for EFA; Sample B used for CFA. χ^2^ = chi-square; t = independent-samples *t*-test. All *p*-values are two-tailed.

**Table 3:** Response Scoring Schema for GAB-A Scales.

Response Category	Schema A	Schema B	Interpretation
	(GRAS)	(GTI)	
Stereotypical	2	2	Traditional gender differentiation:
(matches traditional gender expectation)			assigns trait/role/activity based on gender
Egalitarian	1	0	Rejection of gender categorization:
(“It doesn’t matter”/no gender distinction)			denies relevance of gender to the domain
Counter-stereotypical	0	1	Reversed but still gendered categorization:
(opposite to traditional expectation)			assigns trait/role/activity to the non-traditional gender

Note. Schema A (used for GSAS and GRAS) treats the stereotypical response as the maximum score, with the egalitarian response as the midpoint. Schema B (used for GTI) treats the egalitarian response as the zero-point (absence of gendered categorization), recognizing that counter-stereotypical attributions still reflect engagement with essentialist reasoning, albeit in the non-traditional direction. Higher total scores on all scales indicate greater adherence to gender stereotypes/roles.

**Table 4:** Content Validity Indices by Module.

Module	Items	I-CVI Range	I-CVI = 1.00	I-CVI ≥ 0.90	I-CVI ≥ 0.78	S-CVI/Ave
1. GSAS (Gender Stereotyped Attitude Scale)	18	0.83–1.00	16 (88.9%)	17 (94.4%)	18 (100%)	0.986
2. GRAS (Gender Role Activities Scale)	18	0.83–1.00	14 (77.8%)	17 (94.4%)	18 (100%)	0.977
3. GTI (Gendered Traits Inventory)	10	0.67–1.00	5 (50.0%)	7 (70.0%)	9 (90.0%)	0.917
**Total Scale**	**46**	**0.67–1.00**	**35 (76.1%)**	**41 (89.1%)**	**45 (97.8%)**	**0.967**

Note. I-CVI = Item-level Content Validity Index. S-CVI/Ave = Scale-level Content Validity Index (average method). Expert panel: N = 12.

**Table 5:** GSAS Factor Structure: EFA Pattern Coefficients and CFA Standardized Loadings.

Item	Content (Abbreviated)	EFA F1	EFA F2	EFA F3	CFA λ	h^2^
	***Factor 1: Traditional Gender Stereotypes***					
GSAS_1	Women cares for home	0.75	0.04	−0.04	0.73	0.58
GSAS_2	Woman family > career	0.73	0.03	−0.01	0.72	0.56
GSAS_3	Women stays home if man earns	0.68	−0.05	0.02	0.60	0.44
GSAS_4	Men science, women humanities	0.36	0.17	0.03	0.49	0.24
GSAS_6	Men better leaders	0.43	0.24	0.01	0.67	0.39
GSAS_7	Woman’s role = mother+wife	0.43	0.26	0.03	0.75	0.43
GSAS_9	Women emotionally fragile	0.43	0.08	0.06	0.50	0.26
GSAS_11	Man must protect woman	0.25	0.17	0.12	0.50	0.22
GSAS_18	Mothers > fathers’ childcare	0.32	0.15	0.17	0.63	0.30
	***Factor 2: Violence/Sexuality Myths***					
GSAS_5	Female infidelity worse	0.04	0.70	−0.03	0.71	0.50
GSAS_8	Normal for men to lose temper	0.01	0.64	0.05	0.66	0.46
GSAS_13	IPV is private matter	0.10	0.49	−0.07	0.42	0.26
GSAS_15	“No” means “yes”	−0.03	0.61	0.14	0.58	0.46
GSAS_16	Dress provocatively = harassment	0.10	0.51	0.09	0.64	0.39
	***Factor 3: Relational Control***					
GSAS_12	Man checks partner’s phone	0.03	0.04	0.69	0.71	0.54
GSAS_14	Man has partner’s passwords	−0.02	0.08	0.79	0.73	0.67
GSAS_17	Man knows partner’s location	0.13	0.05	0.51	0.66	0.42

Note. EFA = Exploratory factor analysis (Sample A, n = 1479); CFA = Confirmatory factor analysis (Sample B, n = 1476). EFA used ML extraction with oblimin rotation.. h^2^ = communality. GSAS_10 excluded. All CFA λ *p* < 0.001.

**Table 6:** GSAS (Gender Stereotyped Attitude Scale) Known-Groups Validity.

**Panel A: Gender Differences (N = 2955)**						
**Scale**	**Female M (SD)**	**Male M (SD)**	**t**	***p***	**d [95% CI]**	
Total Scale (17 items)	1.67 (0.42)	2.18 (0.53)	29.71	<0.001	1.07 [0.99, 1.15]	
GSAS-TS: Traditional Stereotypes	1.85 (0.52)	2.39 (0.59)	25.99	<0.001	0.95 [0.87, 1.03]	
GSAS-VM: Violence and Sexuality Myths	1.31 (0.39)	1.87 (0.63)	29.75	<0.001	1.04 [0.97, 1.12]	
GSAS-RC: Relational Control	1.71 (0.61)	2.09 (0.71)	15.33	<0.001	0.56 [0.48, 0.63]	
**Panel B: School Type Differences**						
**Scale**	**Academic M (SD)**	**Technical M (SD)**	**Vocational M (SD)**	**F**	***p***	**η^2^**
Total Scale (17 items)	1.82 (0.52)	2.12 (0.52)	2.03 (0.56)	100.85	<0.001	0.064
GSAS-TS: Traditional Stereotypes	2.00 (0.61)	2.32 (0.59)	2.22 (0.61)	87.18	<0.001	0.056
GSAS-VM: Violence and Sexuality Myths	1.49 (0.53)	1.77 (0.63)	1.72 (0.67)	68.89	<0.001	0.045
GSAS-RC: Relational Control	1.80 (0.65)	2.08 (0.70)	1.97 (0.73)	50.69	<0.001	0.033
**Panel C: Factorial ANOVA (Total Scale)**						
**Effect**	**df**	**F**	***p***		**ηp^2^**	
Gender	1	362.96	<0.001		0.110	
School Type	2	31.35	<0.001		0.021	
Gender × School	2	0.39	0.680		0.000	

Note. Panel A: Female n = 1289; Male n = 1666. Welch’s *t*-test used. Scale range 1–4; higher = greater stereotype endorsement. Panel C: Female n = 1289; Male n = 1666. Welch’s *t*-test used. Scale range 1–4; higher = greater stereotype endorsement.

**Table 7:** GRAS Factor Structure: EFA Pattern Coefficients and CFA Standardized Loadings (14 Items).

Item	Content (Abbreviated)	EFA F1	EFA F2	CFA λ	h^2^
	*Factor 1: Recreational/Expressive Activities*				
GRAS_7	Playing soccer	0.99	-	0.90	0.86
GRAS_14	Playing video games	0.77	-	0.77	0.58
GRAS_8	Dancing	0.75	-	0.79	0.66
GRAS_16	Combat sports	0.70	-	0.76	0.62
GRAS_17	Reading books	0.47	-	0.50	0.27
	*Factor 2: Social/Economic Roles*				
GRAS_10	Earning money	-	0.79	0.76	0.60
GRAS_9	Being in charge at work	-	0.78	0.74	0.57
GRAS_3	Supporting family financially	-	0.72	0.62	0.47
GRAS_12	Being President	-	0.65	0.70	0.49
GRAS_6	Cleaning house	-	0.56	0.69	0.50
GRAS_5	Taking care of children	-	0.42	0.61	0.33
GRAS_18	Being a police officer	-	0.37	0.58	0.34
GRAS_11	Grocery shopping	-	0.35	0.40	0.14
GRAS_2	Cooking	-	-	0.37	0.13

Note. EFA (Sample A, n = 1479) with polychoric correlations and oblimin rotation; CFA (Sample B, n = 1476) using WLSMV. Dashes indicate loadings < 0.30. Scale: 0 = egalitarian, 1 = indifferent, 2 = stereotypical. Items 1, 4, 13, 15 excluded.

**Table 8:** GRAS (Gender Role Activities Scale) Known-Groups Validity.

**Panel A: Gender Differences (N = 2955)**						
**Scale**	**Female M (SD)**	**Male M (SD)**	**t**	***p***	**d [95% CI]**	
Total Scale (14 items)	1.19 (0.22)	1.42 (0.28)	24.55	<0.001	0.88 [0.81, 0.96]	
F1: Recreational/Expressive	1.29 (0.33)	1.59 (0.33)	24.02	<0.001	0.89 [0.81, 0.97]	
F2: Social/Economic Roles	1.13 (0.21)	1.32 (0.31)	19.82	<0.001	0.70 [0.63, 0.78]	
**Panel B: School Type Differences**						
**Scale**	**Academic M (SD)**	**Technical M (SD)**	**Vocational M (SD)**	**F**	***p***	**η^2^**
Total Scale (14 items)	1.29 (0.27)	1.37 (0.29)	1.29 (0.28)	31.89	<0.001	0.021
F1: Recreational/Expressive	1.44 (0.35)	1.52 (0.36)	1.41 (0.36)	24.07	<0.001	0.016
F2: Social/Economic Roles	1.21 (0.27)	1.29 (0.30)	1.22 (0.28)	28.08	<0.001	0.019
**Panel C: Factorial ANOVA (Total Scale)**						
**Effect**	**df**	**F**	***p***		**ηp^2^**	
Gender	1	289.76	<0.001		0.089	
School Type	2	6.08	0.002		0.004	
Gender × School	2	2.13	0.119		0.001	

Note. Gender × School interaction not significant (*p* = 0.119). Scale range 0–2; higher = greater stereotype endorsement.

**Table 9:** GTI Factor Structure: CFA Standardized Loadings and Reliability (Sample B, n = 1476).

Item	Content	Empirical Direction	λ	SE	R^2^
GTI_1	Independence	Male	0.53	0.027	0.28
GTI_2	Aggressiveness	Male	0.70	0.026	0.48
GTI_3	Selfishness	Male	0.67	0.022	0.45
GTI_4	Self-confidence	Male	0.66	0.022	0.44
GTI_5	Sensitivity	Female	0.77	0.022	0.60
GTI_6	Reserve	Female	0.65	0.022	0.43
GTI_7	Unpredictability	Male *	0.62	0.023	0.39
GTI_8	Fragility	Female	0.72	0.022	0.51
GTI_9	Cooperativeness	Female	0.64	0.024	0.41
GTI_10	Reasonableness	Female *	0.63	0.023	0.40

Note. λ = standardized factor loading; all *p* < 0.001. Unidimensional model: χ^2^(35) = 321.56, CFI = 0.948, TLI = 0.934, RMSEA = 0.075, SRMR = 0.063. McDonald’s ω = 0.823. Schema B coding: 0 = egalitarian, 1 = counter-stereotypical, 2 = stereotypical. * Items coded using empirically derived (not theoretically expected) stereotype directions.

**Table 10:** GTI (Gendered Traits Inventory) Known-Groups Validity.

**Panel A: Gender Differences (N = 2955)**						
**Scale**	**Female M (SD)**	**Male M (SD)**	**t**	***p***	**d [95% CI]**	
Total Scale (10 items)	0.78 (0.53)	0.83 (0.53)	2.94	0.003	0.11 [0.04, 0.18]	
**Panel B: School Type Differences**						
**Scale**	**Academic M (SD)**	**Technical M (SD)**	**Vocational M (SD)**	**F**	***p***	**η^2^**
Total Scale (10 items)	0.78 (0.52)	0.85 (0.53)	0.80 (0.56)	4.47	0.012	0.003
**Panel C: Factorial ANOVA (Total Scale)**						
**Effect**	**df**	**F**	***p***		**ηp^2^**	
Gender	1	5.02	0.025		0.002	
School Type	2	1.56	0.211		0.001	
Gender × School	2	0.55	0.579		0.000	

Note. Panel A: Female n = 1289; Male n = 1666. Welch’s *t*-test used. Scale range 0–2; higher = greater stereotype endorsement. Panel C: Gender × School interaction not significant (*p* = 0.579). Scale range 0–2; higher = greater stereotype endorsement.

**Table 11:** Measurement Invariance Across Gender for GAB-A Scales.

**Panel A: Model Fit Indices by Invariance Level**									
**Scale**	**Model**	**Level**	**χ^2^**	**df**	**CFI**	**TLI**	**RMSEA**	**90% CI**	**SRMR**
GSAS	3F	Configural	916.67	232	0.988	0.985	0.045	[0.042, 0.048]	0.047
		Metric	1159.19	246	0.983	0.982	0.05	[0.047, 0.053]	0.052
		Scalar	1345.72	277	0.981	0.981	0.051	[0.048, 0.054]	0.049
	4F	Configural	793.87	226	0.99	0.988	0.041	[0.038, 0.044]	0.044
		Metric	990.52	239	0.986	0.984	0.046	[0.043, 0.049]	0.049
		Scalar	1212.75	269	0.983	0.983	0.049	[0.046, 0.052]	0.047
GRAS	2F (14 items)	Configural	749.57	152	0.986	0.983	0.052	[0.048, 0.055]	0.052
		Metric	1133.52	164	0.977	0.974	0.063	[0.060, 0.067]	0.063
		Scalar	1086.28	176	0.978	0.978	0.059	[0.056, 0.063]	0.055
GTI	1F	Configural	457.76	70	0.978	0.971	0.061	[0.056, 0.067]	0.064
		Metric	483.27	79	0.977	0.974	0.059	[0.054, 0.064]	0.065
		Scalar (full)	795.64	88	0.959	0.958	0.074	[0.069, 0.079]	0.07
		Partial Scalar	-	-	0.971	0.967	0.054	[0.049, 0.059]	0.058
**Panel B: Invariance Tests (Chen, 2007 Criteria)**									
**Scale**	**Model**	**Comparison**		**ΔCFI**		**ΔRMSEA**		**Decision**	
GSAS	3F	Configural → Metric		−0.004		0.005		Pass	
		Metric → Scalar		−0.003		0.001		Pass	
	4F	Configural → Metric		−0.003		0.005		Pass	
		Metric → Scalar		−0.004		0.003		Pass	
GRAS	2F (14 items)	Configural → Metric		−0.009		0.012		Pass	
		Metric → Scalar		0.001		−0.004		Pass	
GTI	1F	Configural → Metric		−0.001		−0.002		Pass	
		Metric → Scalar		−0.018		0.015		Fail	
		Metric → Partial Scalar		−0.006		0.005		Pass	
**Panel C: Invariance Summary**									
**Scale**	**Items**	**Invariance Level**		**DIF Handling**		**Anchor Items**		**d_lat**	**Validity**
GSAS	17	Full Scalar		None		17 (100%)		1.07	Excellent
GRAS	14	Full Scalar		Item 1 removed		14 (100%)		1.06	Excellent
GTI	10	Partial Scalar		2 (freed)		8 (80%)		0.14	Good

Note. Panel A: WLSMV estimation used for all models. N = 2955 (Female n = 1289; Male n = 1666). 3F = three-factor model; 4F = four-factor model; 2F = two-factor model; 1F = one-factor model. GRAS analyses conducted with 14 items (item 1 excluded due to DIF). GTI partial scalar with 8 anchor items. Configural = same factor structure; Metric = equal loadings; Scalar = equal thresholds/intercepts. GTI Partial Scalar model achieved after releasing thresholds for items GTI_1 and GTI_10 (see Table S14). Panel B: Invariance supported when ΔCFI ≥ −0.010 and ΔRMSEA ≤ 0.015 (Chen, 2007). GTI failed full scalar invariance; partial scalar achieved by freeing thresholds for items 1 and 10. Panel C: GRAS item 1 removed from scale due to differential loading (λ = 0.39 females vs. 0.70 males). GTI items 1 (Independence) and 10 (Reasonableness) retained with freed thresholds (partial scalar). d_lat = latent mean difference (Males − Females) in SD units.

**Table 12:** Measurement Invariance Across School Type for GAB-A Scales.

**Panel A: Model Fit Indices by Invariance Level**									
**Scale**	**Model**	**Level**	**χ^2^**	**df**	**CFI**	**TLI**	**RMSEA**	**90% CI**	**SRMR**
GSAS	3F	Configural	889.16	348	0.993	0.992	0.04	[0.037, 0.043]	0.044
GSAS	3F	Metric	1116.63	376	0.991	0.99	0.045	[0.042, 0.048]	0.05
GSAS	3F	Scalar	1164.06	438	0.991	0.991	0.041	[0.038, 0.044]	0.045
GRAS	2F	Configural	1088.96	228	0.987	0.985	0.062	[0.058, 0.066]	0.054
GRAS	2F	Metric	1354.97	252	0.984	0.982	0.067	[0.063, 0.070]	0.06
GRAS	2F	Scalar	1221.75	276	0.986	0.986	0.059	[0.056, 0.062]	0.055
GTI	1F	Configural	498.47	105	0.978	0.971	0.062	[0.056, 0.067]	0.066
GTI	1F	Metric	547.07	123	0.976	0.974	0.059	[0.054, 0.064]	0.068
GTI	1F	Scalar	558.62	141	0.976	0.977	0.055	[0.050, 0.060]	0.066
**Panel B: Invariance Tests**									
**Scale**	**Model**	**Comparison**		**ΔCFI**		**ΔRMSEA**		**Decision**	
GSAS	3F	Configural → Metric		−0.003		0.005		Supported	
GSAS	3F	Metric → Scalar		0		−0.004		Supported	
GRAS	2F	Configural → Metric		−0.004		0.005		Supported	
GRAS	2F	Metric → Scalar		0.002		−0.008		Supported	
GTI	1F	Configural → Metric		−0.002		−0.003		Supported	
GTI	1F	Metric → Scalar		0		−0.004		Supported	
**Panel C: Invariance Summary**									
**Scale**	**Model**	**Items**		**Invariance**		**DIF**	**d(T−A)**	**d(V−A)**	**d(V−T)**
GSAS	3F	17		Scalar		0	0.54	0.35	−0.2
GRAS	2F	14		Scalar		0	0.32	−0.01	−0.33
GTI	1F	10		Scalar		0	0.13	0.02	−0.11

Note. Panel A: N = 2,955 (Academic n = 1390; Technical n = 989; Vocational n = 576). CFI = comparative fit index; TLI = Tucker–Lewis index; RMSEA = root mean square error of approximation; CI = confidence interval; SRMR = standardized root mean square residual. Estimator: WLSMV. Panel B: Chen (2007) criteria for invariance support: ΔCFI ≥ −0.010 and ΔRMSEA ≤ 0.015. All comparisons met both criteria, supporting full scalar invariance across school tracks. Panel C: DIF = number of items showing differential item functioning (χ^2^ > 10 from score test). d = latent mean difference in SD units (weighted average for multifactor scales). T−A = Technical−Academic; V−A = Vocational−Academic; V−T = Vocational−Technical. Full scalar invariance permits valid latent mean comparisons across all groups.

**Table 13:** Convergent and Discriminant Validity Evidence for GAB-A Scales.

**Panel A: Correlations with Criterion Measures (N = 2955)**								
**c**	**BPAQ Tot**	**BPAQ Physical**	**BPAQ Verbal**	**BPAQ Anger**	**BPAQ Hostility**	**K10 Distress**	**RSES Self-Esteem**	**PAQ-S**
GSAS Total	0.047 *	0.250 ***	−0.057 **	−0.024	−0.101 ***	−0.149 ***	0.123 ***	−0.096 ***
GSAS-TS	0.026	0.220 ***†	−0.056 **†	−0.039 *†	−0.111 ***†	−0.155 ***†	0.133 ***†	−0.097 ***†
GSAS-VM	0.002	0.191 ***†	−0.103 ***†	−0.058 **†	−0.103 ***†	−0.150 ***†	0.104 ***†	−0.098 ***†
GSAS-RC	0.133 ***†	0.241 ***†	0.048 **†	0.084 ***†	−0.001	−0.027	0.036	−0.024
GRAS Total	0.027	0.187 ***	−0.018	−0.026	−0.106 ***	−0.184 ***	0.161 ***	−0.113 ***
GRAS-SR Social Roles	−0.005	0.141 ***†	−0.019	−0.051 **†	−0.122 ***†	−0.210 ***†	0.169 ***†	−0.121 ***†
GRAS-LA Leisure Activities	0.044 *†	0.186 ***†	−0.014	−0.004	−0.077 ***†	−0.134 ***†	0.126 ***†	−0.088 ***†
GTI Total	0.189 ***†	0.178 ***†	0.113 ***†	0.156 ***†	0.132 ***†	0.073 ***†	−0.045 *†	0.102 ***†
**Panel B: Inter-Scale Correlations**								
	**G26 Tot**	**G26 F1**	**G26 F2**	**G26 F3**	**G27 Tot**	**G27 F1**	**G27 F2**	**G29 Tot**
GSAS	-							
GSAS-TS	0.940 ***	-						
GSAS-VM	0.843 ***	0.676 ***†	-					
GSAS-RC	0.683 ***	0.502 ***†	0.467 ***†	-				
GRAS	0.653 ***	0.667 ***	0.519 ***	0.350 ***	-			
GRAS-LA	0.521 ***	0.528 ***†	0.418 ***†	0.284 ***†	0.846 ***	-		
GRAS-SR	0.628 ***	0.644 ***†	0.498 ***†	0.333 ***†	0.930 ***	0.590 ***†	-	
GTI	0.312 ***	0.337 ***†	0.210 ***†	0.175 ***†	0.440 ***	0.403 ***†	0.388 ***†	-

Note. Panel A: * *p* < 0.05, ** *p* < 0.01, *** *p* < 0.001 (uncorrected); † significant after FDR correction (α = 0.05). GSAS = Gender Stereotyped Attitude Scale; GRAS = Gender Role Activities Scale; GTI = Gendered Traits Inventory. GSAS-TS = Traditional Stereotypes; GSAS-VM = Violence/Sexuality Myths; GSAS-RC = Relational Control; GRAS-LA = Leisure Activities; GRAS-SR = Social Roles. BPAQ = Buss-Perry Aggression Questionnaire; K10 = Kessler Psychological Distress Scale; RSES = Rosenberg Self-Esteem Scale; PAQ-S = Perth Alexithymia Questionnaire—Short Form. * *p* < 0.05. ** *p* < 0.01. *** *p* < 0.001. † Subscale correlation. Panel B: * *p* < 0.05, ** *p* < 0.01, *** *p* < 0.001; † FDR corrected.

**Table 14:** Sensitivity Analysis: Impact of Excluding Problematic Cases.

Scale	Sample	n	Excluded	%	M	SD	α	d_Gender	ΔM	Δα	Δd
GSAS (GSAS)	Full	2955	0	0.00	1.959	0.544	0.894	−1.07	-	-	-
GSAS (GSAS)	Excluding Problematic	2911	44	1.49	1.964	0.531	0.887	−1.08	0.006	−0.007	−0.01
GRAS (GRAS)	Full	2955	0	0.00	1.318	0.28	0.850	−0.88	-	-	-
GRAS (GRAS)	Excluding Problematic	2954	1	0.03	1.318	0.28	0.850	−0.88	0.000	0.000	0.00
GTI (GTI)	Full	2955	0	0.00	0.809	0.534	0.802	−0.11	-	-	-
GTI (GTI)	Excluding Problematic	2950	5	0.17	0.808	0.534	0.802	−0.11	−0.001	0.000	0.00

Note. GSAS = Gender Stereotyped Attitude Scale; GRAS = Gender Role Activities Scale; GTI = Gendered Traits Inventory. d_gender = Cohen’s d for gender differences (positive = females higher). Δ = change from full sample. α = Cronbach’s alpha.

## Data Availability

The data presented in this study are available upon request from the corresponding author due to privacy restrictions.

## Supplementary Materials

The following supporting information can be downloaded at https://www.mdpi.com/article/10.3390/bs16030413/s1. File S1: Content validity documentation and item development procedures. File S2: Factor structure details for the GSAS, GRAS, and GTI (EFA and CFA results). File S3: Reliability indices and item-level analyses. File S4: Measurement invariance analyses across gender. File S5: Item-level gender differences. File S6: Criterion validity analyses. File S7: Response styles analyses. File S8: Gender-stratified normative data and cut-off scores. File S9: Full item content and response frequencies. Files S10–S13: Administration and scoring forms for the GAB-A modules.
