# Supplementary material for: The GAB-A: Development and Validation of the Gender Stereotypes and Roles Adherence Battery for Adolescents
Source: Behav Sci (Basel). 2026 Mar 11;16(3):413. doi: 10.3390/bs16030413 (PMC13024607; doi:10.3390/bs16030413)
Supplement: Supplementary file 1 [file behavsci-16-00413-s001.zip › GAB-A_Supplementary_S13_Manuale_Scoring_IT.pdf]

GAB-A

Gender Stereotypes and Roles Adherence Battery for Adolescents

Batteria di Adesione a Stereotipi e Ruoli di Genere per Adolescenti

MANUALE DI SCORING E INTERPRETAZIONE

Antonio Tintori[1], Giulia Ciancimino[1]*, David Vagni[2], Loredana Cerbara[1]

[1] *Istituto di Ricerche sulla Popolazione e le Politiche Sociali, Consiglio Nazionale delle Ricerche, Roma, Italia*

[2] *Istituto di Ricerca e Innovazione Biomedica, Consiglio Nazionale delle Ricerche, Roma, Italia*

* *Corrispondenza: giulia.ciancimino@irpps.cnr.it*

© 2026 CNR-IRPPS

1. Panoramica della Batteria

La GAB-A è composta da tre scale psicometricamente validate:

Scala	Nome completo	Item	α
**GSAS**	Scala di Atteggiamenti Stereotipati di Genere	17	.89
**GRAS**	Scala di Attività e Ruoli di Genere	14	.85
**GTI**	Inventario dei Tratti di Genere	10	.80

Campione normativo: N = 2.955 adolescenti italiani in prima superiore (età 14-15 anni), scuole secondarie di II grado di Roma.

2. Algoritmo di Scoring: GSAS

2.1 Codifica delle risposte

La GSAS utilizza una scala Likert a 4 punti. Tutti i 17 item sono codificati nella stessa direzione:

Risposta	Punteggio
Molto in disaccordo	1
Abbastanza in disaccordo	2
Abbastanza d'accordo	3
Molto d'accordo	4

2.2 Calcolo dei punteggi

Punteggio somma totale: Sommare tutti i 17 item. Range: 17-68.

Punteggio medio: Dividere il punteggio somma per 17. Range: 1.00-4.00.

Sottoscale GSAS

Sottoscala	Item (n° somm.)	Range	Range M	α
**GSAS-TS** Stereotipi Tradizionali	1, 2, 3, 4, 6, 7, 9, 10, 17	9-36	1.00-4.00	.85
**GSAS-VM** Miti Violenza/Sessualità	5, 8, 12, 14, 15	5-20	1.00-4.00	.74
**GSAS-RC** Controllo Relazionale	11, 13, 16	3-12	1.00-4.00	.74

3. Algoritmo di Scoring: GRAS

3.1 Ricodifica delle risposte

La GRAS richiede ricodifica in base alla **direzione stereotipica** di ciascun item:

Schema di codifica: 2 = Stereotipico | 1 = Egualitario | 0 = Contro-stereotipico

Item GRAS	Stereotipo	M →	F →	I →
1. Cucinare	**F**	0	2	1
2. Mantenere economicamente la famiglia	**M**	2	0	1
3. Occuparsi dei figli	**F**	0	2	1
4. Pulire casa	**F**	0	2	1
5. Giocare a calcio	**M**	2	0	1
6. Danzare	**F**	0	2	1
7. Comandare a lavoro	**M**	2	0	1
8. Guadagnare tanti soldi	**M**	2	0	1
9. Fare la spesa	**F**	0	2	1
10. Fare il Presidente	**M**	2	0	1
11. Giocare ai videogiochi	**M**	2	0	1
12. Fare sport da combattimento	**M**	2	0	1
13. Leggere libri	**F**	0	2	1
14. Fare il poliziotto	**M**	2	0	1

3.2 Calcolo dei punteggi

Punteggio somma totale: Sommare tutti i 14 item ricodificati. Range: 0-28.

Punteggio medio: Dividere il punteggio somma per 14. Range: 0.00-2.00.

Sottoscale GRAS

Sottoscala	Item (n° somm.)	Range	Range M	α
**GRAS-LA** Attività Tempo Libero	5, 6, 11, 12, 13	0-10	0.00-2.00	.78
**GRAS-SR** Ruoli Sociali	1, 2, 3, 4, 7, 8, 9, 10, 14	0-18	0.00-2.00	.78

4. Algoritmo di Scoring: GTI

4.1 Ricodifica delle risposte

La GTI richiede ricodifica in base alla direzione stereotipica empiricamente validata:

Schema di codifica GTI: 2 = Stereotipico | 1 = Contro-stereotipico | 0 = Egualitario

Item GTI	Stereotipo	M →	F →	I →
1. Indipendenza	**M**	2	1	0
2. Aggressività	**M**	2	1	0
3. Egoismo	**M**	2	1	0
4. Fiducia in sé	**M**	2	1	0
5. Sensibilità	**F**	1	2	0
6. Riservatezza	**F**	1	2	0
7. Imprevedibilità	**M**	2	1	0
8. Fragilità	**F**	1	2	0
9. Collaboratività	**F**	1	2	0
10. Ragionevolezza	**F**	1	2	0

4.2 Calcolo dei punteggi

Punteggio somma totale: Sommare tutti i 10 item ricodificati. Range: 0-20.

Punteggio medio: Dividere il punteggio somma per 10. Range: 0.00-2.00.

Nota: GTI è unidimensionale. Non sono previste sottoscale.

5. Tabelle Normative

Campione normativo: N = 2.955 (Femmine: 1.289 | Maschi: 1.666)

5.1 Distribuzione Percentile (Punteggi Somma)

GSAS – Scala di Atteggiamenti Stereotipati di Genere

Scala	Gruppo	P5	P10	P25	P50	P75	P90	P95
Totale (17)	Totale	20	22	27	32	38	43	46
	Femmine	19	20	23	28	33	38	41
	Maschi	22	25	31	37	43	48	51
GSAS-TS (9)	Totale	10	12	14	18	22	24	26
	Femmine	10	11	13	16	20	23	25
	Maschi	12	14	18	22	25	28	30
GSAS-VM (5)	Totale	5	5	6	8	10	12	13
	Femmine	5	5	5	6	7	9	10
	Maschi	5	5	7	9	12	14	16
GSAS-RC (3)	Totale	4	4	6	6	7	8	9
	Femmine	3	3	3	4	6	8	8
	Maschi	3	3	4	6	8	10	11

GRAS e GTI

Scala	Gruppo	P5	P10	P25	P50	P75	P90	P95
GRAS Tot (14)	Totale	14	14	16	18	21	23	24
	Femmine	14	14	14	16	19	21	23
	Maschi	14	14	17	20	23	25	26
GTI Tot (10)	Totale	0	0	4	8	12	15	17
	Femmine	0	0	4	8	12	15	17
	Maschi	0	0	4	8	12	15	17

6. Cut-off Empirici Validati

I cut-off sono stati validati empiricamente combinando l'analisi Natural Breaks (KDE) con la distribuzione percentilica:

6.1 GSAS - Classificazione Combinata

Categoria	Range Somma	Range Media	Percentile	% Tot	Livello
Basso	17-25	1.00-1.47	P0-P25	25%	Normale
Medio-Basso	26-35	1.53-2.06	P26-P63	38%	Normale
Medio-Alto	36-37	2.12-2.18	P64-P73	7%	Attenzione
**Elevato**	38-42	2.24-2.47	P74-P89	15%	**Elevato**
**Molto Elevato**	**≥43**	**≥2.53**	**P90-P100**	**17%**	**Allerta**

6.2 Cut-off per Scala (Punteggi Somma)

Scala	Gruppo	Elevato ≥	% ≥	Allerta ≥	% ≥
GSAS Totale	Totale	38	30%	43	17%
	Femmine	38	11%	43	4%
	Maschi	38	49%	43	28%
GSAS-TS	Totale	22	28%	24	18%
GSAS-VM	Totale	10	29%	12	13%
GSAS-RC	Totale	7	40%	8	24%
GRAS Totale	Totale	21	28%	23	17%
	Femmine	21	13%	23	6%
	Maschi	21	42%	23	28%
GTI Totale	Totale	12	28%	15	12%

7. Guida all'Interpretazione

7.1 Differenze di genere attese

La letteratura e i dati normativi evidenziano differenze sistematiche:

- **GSAS:** I maschi mostrano punteggi significativamente più elevati ($d \approx 1.0$, differenza ampia)
- **GRAS:** I maschi mostrano punteggi moderatamente più elevati ($d \approx 0.7$)
- **GTI:** Differenze di genere minime ($d \approx 0.1$) - interpretare con norme totali

7.2 Considerazioni cliniche

Punteggi elevati nelle scale GSAS-VM (Miti su Violenza/Sessualità) e GSAS-RC (Controllo Relazionale) meritano particolare attenzione, poiché associati a:

- Atteggiamenti più tolleranti verso la violenza di genere
- Maggiore propensione a comportamenti di controllo relazionale
- Correlazioni positive con aggressività (fisica, verbale, ostilità)

Nota metodologica: La batteria GAB-A ha raggiunto l'invarianza scalare per genere e tipo di scuola, permettendo confronti validi tra gruppi.

© 2026 CNR-IRPPS. Tutti i diritti riservati.
Per uso esclusivo di ricerca. La riproduzione è consentita con citazione della fonte.
