# Supplementary material for: The GAB-A: Development and Validation of the Gender Stereotypes and Roles Adherence Battery for Adolescents
Source: Behav Sci (Basel). 2026 Mar 11;16(3):413. doi: 10.3390/bs16030413 (PMC13024607; doi:10.3390/bs16030413)
Supplement: Supplementary file 1 [file behavsci-16-00413-s001.zip › GAB-A_Supplementary_S10_Administration_Form_EN.pdf]

GAB-A

Gender Stereotypes and Roles Adherence Battery for Adolescents

ADMINISTRATION FORM

Antonio Tintori[1], Giulia Ciancimino[1]*, David Vagni[2], Loredana Cerbara[1]

[1] *Institute for Research on Population and Social Policies, National Research Council of Italy, Rome, Italy*

[2] *Institute for Research and Innovation in Biomedicine, National Research Council of Italy, Rome, Italy*

* *Correspondence: giulia.ciancimino@irpps.cnr.it*

© 2026 CNR-IRPPS

Administration Instructions

The GAB-A battery consists of three independent scales that can be administered together or separately:

GSAS – Gender Stereotyped Attitude Scale (17 items, 4-point Likert scale)

GRAS – Gender Role Activities Scale (14 items, categorical response)

GTI – Gendered Traits Inventory (10 items, categorical response)

Estimated administration time: 10-15 minutes for the complete battery.

Demographic Information

Age: _____ years

Gender: □ Male □ Female □ Other / Prefer not to answer

Grade: _____ School type: □ Academic □ Technical □ Vocational

Date: ___ / ___ / ______

SCALE 1: GSAS – Gender Stereotyped Attitude Scale

Instructions: Thinking about the relationship between men and women, indicate how much you agree with the following statements.

Response options: 1 = Strongly disagree | 2 = Somewhat disagree | 3 = Somewhat agree | 4 = Strongly agree

Statement	1	2	3	4
1. It is right that the woman should take care of the house	☐	☐	☐	☐
2. A woman should focus more on family and less on her career	☐	☐	☐	☐
3. It is right for a woman to stay home if the man earns enough for both	☐	☐	☐	☐
4. Men are better suited for scientific subjects and women for humanities	☐	☐	☐	☐
5. In a couple, female infidelity is more serious than male infidelity	☐	☐	☐	☐
6. Men have greater leadership ability than women	☐	☐	☐	☐
7. A woman's main role is that of mother and wife	☐	☐	☐	☐
8. It is normal for a man to lose his temper more than a woman	☐	☐	☐	☐
9. Women are emotionally more fragile than men	☐	☐	☐	☐
10. A man has the duty to protect the woman	☐	☐	☐	☐
11. It is right for a man to check his partner's phone	☐	☐	☐	☐
12. Intimate partner violence is a private matter that others should not interfere with	☐	☐	☐	☐
13. It is right for a man to have his partner's social media passwords	☐	☐	☐	☐
14. When women say 'no' to sex, they actually want to do it	☐	☐	☐	☐
15. To avoid sexual harassment, women should not dress provocatively	☐	☐	☐	☐
16. It is right for a man to always know where his partner is	☐	☐	☐	☐
17. It is appropriate for mothers to take care of children more than fathers	☐	☐	☐	☐

SCALE 2: GRAS – Gender Role Activities Scale

Instructions: In your opinion, who is better suited for the following activities?

Response options: M = Males | F = Females | I = It doesn't matter

Activity	M	F	I
1. Cooking	☐	☐	☐
2. Financially supporting the family	☐	☐	☐
3. Taking care of children	☐	☐	☐
4. Cleaning the house	☐	☐	☐
5. Playing soccer*	☐	☐	☐
6. Dancing	☐	☐	☐
7. Being in charge at work	☐	☐	☐
8. Earning a lot of money	☐	☐	☐
9. Grocery shopping	☐	☐	☐
10. Being President	☐	☐	☐
11. Playing video games	☐	☐	☐
12. Practicing combat sports	☐	☐	☐
13. Reading books	☐	☐	☐
14. Being a police officer	☐	☐	☐

**Note: In the original Italian version, this item refers to association football ("calcio").*

SCALE 3: GTI – Gendered Traits Inventory

Instructions: In your opinion, by nature, who is more characterized by:

Response options: M = Males | F = Females | I = It doesn't matter

Personality trait	M	F	I
1. Independence	☐	☐	☐
2. Aggressiveness	☐	☐	☐
3. Selfishness	☐	☐	☐
4. Self-confidence	☐	☐	☐
5. Sensitivity	☐	☐	☐
6. Reserve	☐	☐	☐
7. Unpredictability	☐	☐	☐
8. Fragility	☐	☐	☐
9. Cooperativeness	☐	☐	☐
10. Reasonableness	☐	☐	☐

© 2026 CNR-IRPPS. All rights reserved.
For research use only. Reproduction permitted with citation of source.
