# Supplementary material for: The GAB-A: Development and Validation of the Gender Stereotypes and Roles Adherence Battery for Adolescents
Source: Behav Sci (Basel). 2026 Mar 11;16(3):413. doi: 10.3390/bs16030413 (PMC13024607; doi:10.3390/bs16030413)
Supplement: Supplementary file 1 [file behavsci-16-00413-s001.zip › GAB-A_Supplementary_S11_Scoring_Manual_EN.pdf]

GAB-A

Gender Stereotypes and Roles Adherence Battery for Adolescents

SCORING AND INTERPRETATION MANUAL

Antonio Tintori[1], Giulia Ciancimino[1]*, David Vagni[2], Loredana Cerbara[1]

[1] *Institute for Research on Population and Social Policies, National Research Council of Italy, Rome, Italy*

[2] *Institute for Research and Innovation in Biomedicine, National Research Council of Italy, Rome, Italy*

* *Correspondence: giulia.ciancimino@irpps.cnr.it*

© 2026 CNR-IRPPS

1. Battery Overview

The GAB-A consists of three psychometrically validated scales:

Scale	Full Name	Items	α
**GSAS**	Gender Stereotyped Attitude Scale	17	.89
**GRAS**	Gender Role Activities Scale	14	.85
**GTI**	Gendered Traits Inventory	10	.80

Normative sample: N = 2,955 Italian adolescents in 9th grade (ages 14-15), secondary schools in Rome.

2. Scoring Algorithm: GSAS

2.1 Response Coding

The GSAS uses a 4-point Likert scale. All 17 items are coded in the same direction:

Response	Score
Strongly disagree	1
Somewhat disagree	2
Somewhat agree	3
Strongly agree	4

2.2 Score Calculation

Sum score: Sum all 17 items. Range: 17-68.

Mean score: Divide sum score by 17. Range: 1.00-4.00.

GSAS Subscales

Subscale	Items (admin #)	Range	Mean R	α
**GSAS-TS** Traditional Stereotypes	1, 2, 3, 4, 6, 7, 9, 10, 17	9-36	1.00-4.00	.85
**GSAS-VM** Violence/Sexuality Myths	5, 8, 12, 14, 15	5-20	1.00-4.00	.74
**GSAS-RC** Relational Control	11, 13, 16	3-12	1.00-4.00	.74

3. Scoring Algorithm: GRAS

3.1 Response Recoding

GRAS requires recoding based on the **stereotypical direction** of each item:

Coding Schema A (GRAS): 2 = Stereotypical | 1 = Egalitarian | 0 = Counter-stereotypical

GRAS Item	Stereotype	M →	F →	I →
1. Cooking	**F**	0	2	1
2. Financially supporting the family	**M**	2	0	1
3. Taking care of children	**F**	0	2	1
4. Cleaning the house	**F**	0	2	1
5. Playing soccer	**M**	2	0	1
6. Dancing	**F**	0	2	1
7. Being in charge at work	**M**	2	0	1
8. Earning a lot of money	**M**	2	0	1
9. Grocery shopping	**F**	0	2	1
10. Being President	**M**	2	0	1
11. Playing video games	**M**	2	0	1
12. Practicing combat sports	**M**	2	0	1
13. Reading books	**F**	0	2	1
14. Being a police officer	**M**	2	0	1

3.2 Score Calculation

Sum score: Sum all 14 recoded items. Range: 0-28.

Mean score: Divide sum score by 14. Range: 0.00-2.00.

GRAS Subscales

Subscale	Items (admin #)	Range	Mean R	α
**GRAS-LA** Leisure Activities	5, 6, 11, 12, 13	0-10	0.00-2.00	.78
**GRAS-SR** Social Roles	1, 2, 3, 4, 7, 8, 9, 10, 14	0-18	0.00-2.00	.78

4. Scoring Algorithm: GTI

4.1 Response Recoding

GTI requires recoding based on the empirically validated stereotypical direction:

Coding Schema B (GTI): 2 = Stereotypical | 1 = Counter-stereotypical | 0 = Egalitarian

Note: Schema B differs from GRAS (Schema A). In GTI, the egalitarian response ("It doesn't matter") is scored 0, reflecting minimal engagement with gender categorization, while counter-stereotypical responses score 1.

GTI Item	Stereotype	M →	F →	I →
1. Independence	**M**	2	1	0
2. Aggressiveness	**M**	2	1	0
3. Selfishness	**M**	2	1	0
4. Self-confidence	**M**	2	1	0
5. Sensitivity	**F**	1	2	0
6. Reserve	**F**	1	2	0
7. Unpredictability	**M**	2	1	0
8. Fragility	**F**	1	2	0
9. Cooperativeness	**F**	1	2	0
10. Reasonableness	**F**	1	2	0

4.2 Score Calculation

Sum score: Sum all 10 recoded items. Range: 0-20.

Mean score: Divide sum score by 10. Range: 0.00-2.00.

Note: GTI is unidimensional. No subscales are provided.

5. Normative Tables

Normative sample: N = 2,955 (Females: 1,289 | Males: 1,666)

5.1 Percentile Distribution (Sum Scores)

GSAS - Gender Stereotyped Attitude Scale

Scale	Group	P5	P10	P25	P50	P75	P90	P95
Total (17)	Total	20	22	27	32	38	43	46
	Females	19	20	23	28	33	38	41
	Males	22	25	31	37	43	48	51
GSAS-TS (9)	Total	10	12	14	18	22	24	26
	Females	10	11	13	16	20	23	25
	Males	12	14	18	22	25	28	30
GSAS-VM (5)	Total	5	5	6	8	10	12	13
	Females	5	5	5	6	7	9	10
	Males	5	5	7	9	12	14	16
GSAS-RC (3)	Total	4	4	6	6	7	8	9
	Females	3	3	3	4	6	8	8
	Males	3	3	4	6	8	10	11

GRAS and GTI

Scale	Group	P5	P10	P25	P50	P75	P90	P95
GRAS Tot (14)	Total	14	14	16	18	21	23	24
	Females	14	14	14	16	19	21	23
	Males	14	14	17	20	23	25	26
GTI Tot (10)	Total	0	0	4	8	12	15	17
	Females	0	0	4	8	12	15	17
	Males	0	0	4	8	12	15	17

6. Empirically Validated Cutoffs

Cutoffs were empirically validated by combining Natural Breaks (KDE) analysis with percentile distribution:

6.1 GSAS - Combined Classification

Category	Sum Range	Mean Range	Percentile	% Total	Level
Low	17-25	1.00-1.47	P0-P25	25%	Normal
Medium-Low	26-35	1.53-2.06	P26-P63	38%	Normal
Medium-High	36-37	2.12-2.18	P64-P73	7%	Caution
**Elevated**	38-42	2.24-2.47	P74-P89	15%	**Elevated**
**Very Elevated**	**≥43**	**≥2.53**	**P90-P100**	**17%**	**Alert**

6.2 Cutoffs by Scale (Sum Scores)

Scale	Group	Elevated ≥	% ≥	Alert ≥	% ≥
GSAS Total	Total	38	30%	43	17%
	Females	38	11%	43	4%
	Males	38	49%	43	28%
GSAS-TS	Total	22	28%	24	18%
GSAS-VM	Total	10	29%	12	13%
GSAS-RC	Total	7	40%	8	24%
GRAS Total	Total	21	28%	23	17%
	Females	21	13%	23	6%
	Males	21	42%	23	28%
GTI Total	Total	12	28%	15	12%

7. Interpretation Guide

7.1 Expected Gender Differences

The literature and normative data show systematic differences:

- **GSAS:** Males show significantly higher scores (d ≈ 1.0, large difference)
- **GRAS:** Males show moderately higher scores (d ≈ 0.7)
- **GTI:** Minimal gender differences (d ≈ 0.1) - interpret with total norms

7.2 Clinical Considerations

Elevated scores on GSAS-VM (Violence/Sexuality Myths) and GSAS-RC (Relational Control) subscales warrant particular attention, as they are associated with:

- More tolerant attitudes toward gender-based violence
- Greater propensity for relational control behaviors
- Positive correlations with aggression (physical, verbal, hostility)

Methodological note: The GAB-A battery achieved scalar measurement invariance across gender and school type, allowing valid group comparisons.

© 2026 CNR-IRPPS. All rights reserved.
For research use only. Reproduction permitted with citation of source.
