# Supplementary material for: The GAB-A: Development and Validation of the Gender Stereotypes and Roles Adherence Battery for Adolescents
Source: Behav Sci (Basel). 2026 Mar 11;16(3):413. doi: 10.3390/bs16030413 (PMC13024607; doi:10.3390/bs16030413)
Supplement: Supplementary file 1 [file behavsci-16-00413-s001.zip › GAB-A_Supplementary_S12_Foglio_Somministrazione_IT.pdf]

GAB-A

Gender Stereotypes and Roles Adherence Battery for Adolescents

Batteria di Adesione a Stereotipi e Ruoli di Genere per Adolescenti

FOGLIO DI SOMMINISTRAZIONE

Antonio Tintori[1], Giulia Ciancimino[1]*, David Vagni[2], Loredana Cerbara[1]

[1] *Istituto di Ricerche sulla Popolazione e le Politiche Sociali, Consiglio Nazionale delle Ricerche, Roma, Italia*

[2] *Istituto di Ricerca e Innovazione Biomedica, Consiglio Nazionale delle Ricerche, Roma, Italia*

* *Corrispondenza: giulia.ciancimino@irpps.cnr.it*

© 2026 CNR-IRPPS

Istruzioni per la Somministrazione

La batteria GAB-A è composta da tre scale indipendenti che possono essere somministrate insieme o separatamente:

GSAS – Scala di Atteggiamenti Stereotipati di Genere (17 item, scala Likert 1-4)

GRAS – Scala di Attività e Ruoli di Genere (14 item, risposta categoriale)

GTI – Inventario dei Tratti di Genere (10 item, risposta categoriale)

Tempo di somministrazione stimato: 10-15 minuti per l'intera batteria.

Dati Anagrafici

Età: _____ anni

Genere: □ Maschio □ Femmina □ Altro / Preferisco non rispondere

Classe: _____ Tipo di scuola: □ Liceo □ Istituto Tecnico □ Istituto Professionale

Data: ___ / ___ / ______

SCALA 1: GSAS – Scala di Atteggiamenti Stereotipati di Genere

Istruzioni: Pensando al rapporto tra uomini e donne, indica quanto sei d'accordo con le seguenti affermazioni.

Opzioni di risposta: 1 = Molto in disaccordo | 2 = Abbastanza in disaccordo | 3 = Abbastanza d'accordo | 4 = Molto d'accordo

Affermazione	1	2	3	4
1. È giusto che sia la donna a badare alla casa	☐	☐	☐	☐
2. La donna dovrebbe pensare più alla famiglia e meno alla carriera	☐	☐	☐	☐
3. È giusto che la donna resti a casa se l'uomo guadagna a sufficienza per entrambi	☐	☐	☐	☐
4. Gli uomini sono più portati per le materie scientifiche e le donne per quelle umanistiche	☐	☐	☐	☐
5. Nella coppia, il tradimento femminile è più grave di quello maschile	☐	☐	☐	☐
6. Gli uomini hanno maggiore capacità di leadership delle donne	☐	☐	☐	☐
7. Il ruolo principale della donna è quello di madre e moglie	☐	☐	☐	☐
8. È normale che l'uomo perda la pazienza più della donna	☐	☐	☐	☐
9. La donna è emotivamente più fragile dell'uomo	☐	☐	☐	☐
10. L'uomo ha il compito di proteggere la donna	☐	☐	☐	☐
11. È giusto che l'uomo controlli il cellulare della partner	☐	☐	☐	☐
12. La violenza in coppia è un fatto privato in cui non bisogna intromettersi	☐	☐	☐	☐
13. È giusto che l'uomo abbia le password dei social della partner	☐	☐	☐	☐
14. Quando le donne dicono 'no' al sesso in realtà vorrebbero farlo	☐	☐	☐	☐
15. Per evitare molestie sessuali le donne non dovrebbero vestire in modo provocatorio	☐	☐	☐	☐
16. È giusto che l'uomo sappia sempre dove si trovi la partner	☐	☐	☐	☐
17. È corretto che le madri si occupino dei figli più dei padri	☐	☐	☐	☐

SCALA 2: GRAS – Scala di Attività e Ruoli di Genere

Istruzioni: Secondo te, chi è più portato per le seguenti attività?

Opzioni di risposta: M = Maschi | F = Femmine | I = È indifferente

Attività	M	F	I
1. Cucinare	☐	☐	☐
2. Mantenere economicamente la famiglia	☐	☐	☐
3. Occuparsi dei figli	☐	☐	☐
4. Pulire casa	☐	☐	☐
5. Giocare a calcio	☐	☐	☐
6. Danzare	☐	☐	☐
7. Comandare a lavoro	☐	☐	☐
8. Guadagnare tanti soldi	☐	☐	☐
9. Fare la spesa	☐	☐	☐
10. Fare il Presidente	☐	☐	☐
11. Giocare ai videogiochi	☐	☐	☐
12. Fare sport da combattimento	☐	☐	☐
13. Leggere libri	☐	☐	☐
14. Fare il poliziotto	☐	☐	☐

SCALA 3: GTI – Inventario dei Tratti di Genere

Istruzioni: Secondo te, per sua natura, chi è più caratterizzato da:

Opzioni di risposta: M = Maschi | F = Femmine | I = È indifferente

Tratto di personalità	M	F	I
1. Indipendenza	☐	☐	☐
2. Aggressività	☐	☐	☐
3. Egoismo	☐	☐	☐
4. Fiducia in sé	☐	☐	☐
5. Sensibilità	☐	☐	☐
6. Riservatezza	☐	☐	☐
7. Imprevedibilità	☐	☐	☐
8. Fragilità	☐	☐	☐
9. Collaboratività	☐	☐	☐
10. Ragionevolezza	☐	☐	☐

© 2026 CNR-IRPPS. Tutti i diritti riservati.
Per uso esclusivo di ricerca. La riproduzione è consentita con citazione della fonte.
